# Childbearing Across Immigrants and Their Descendants in Sweden: The Role of Generation and Gender

**DOI:** 10.1177/01979183241245072

**Published:** 2024-04-25

**Authors:** Andreas Höhn, Gunnar Andersson, Hill Kulu, Brad Campbell

**Affiliations:** MRC/CSO Social and Public Health Sciences Unit, 3526 University of Glasgow, Glasgow, UK; School of Geography and Sustainable Development, 7486 University of St Andrews, St Andrews, UK; 7675 Stockholm University Demography Unit (SUDA), Department of Sociology, Stockholm University, Stockholm, Sweden; School of Geography and Sustainable Development, 7486 University of St Andrews, St Andrews, UK; School of Geography and Sustainable Development, University of St Andrews, St Andrews, UK

**Keywords:** fertility, migration, descendants of immigrants, generation 2.5, Sweden

## Abstract

Immigrants and their descendants increasingly shape fertility patterns in European societies. While childbearing among immigrants is well explored, less is known with respect to their descendants. Using Swedish register data, we studied differences in fertility outcomes between first- and second-generation individuals in Sweden and compared with the native Swedish population. We studied men and women separately, distinguished between high- and low-fertility backgrounds, and differentiated whether the descendants of immigrants were offspring from endogamous or exogamous relationships. For most migrants who arrived in Sweden as adults, we found elevated first birth rates shortly after arrival. First birth rates among the second generation were generally close to but lower than the rates observed among native Swedes. Male offspring from exogamous unions with a Swedish-born mother tended to have less depressed rates of first birth than other second-generation individuals. Second birth rates were very similar across population subgroups but generally lower among immigrants and their descendants compared to native Swedes. Third birth rates were often polarized into high- and low-fertility backgrounds, when compared to native Swedes. While fertility patterns among the second generation appeared to drift away from patterns of the first generation, the second generation remained a heterogeneous population subgroup. Nevertheless, and as childbearing patterns of the descendants with one immigrant parent increasingly resembled patterns of native Swedes, exogamous partnerships can likely be considered an important factor behind this gradual family-demographic assimilation process.

## Introduction

As most European countries, Sweden has experienced a growth of its immigrant population within the past decades. According to Statistics Sweden, approximately 20% of Sweden's population in 2023 was foreign-born, while this proportion was less than 7% in 1970 (SCB 2024). Statistics also show that the proportion of newborn children with either one or two foreign-born parents has increased from 16% in 1970 to 38% in 2018 (SCB 2020). The growth of immigrant populations in Western societies has stimulated diverse interdisciplinary research examining differences in demographic behavior between immigrants and their descendants in comparison to the native population.

Studies of fertility among immigrants and their descendants are typically motivated by two factors. First, from a social policy perspective, it is imperative to better understand the childbearing behavior of immigrants as their characteristics have an impact on population structures at local, regional, and national levels (Castles, de Haas and Miller 2013). Unlike other European countries, Sweden has had relatively high levels of fertility. In combination with its positive net migration, Sweden has experienced population growth within the past decades, which is projected to continue in the future (Gassen and Heleniak 2016). Notwithstanding population growth, Sweden is experiencing population aging and the youthful nature of its immigrant populations is likely to counteract this demographic change.

Second, migrant fertility is often considered to reflect some degree of integration into mainstream society and its social fabric (Kulu et al. 2019, Wilson 2019, Mussino, Wilson and Andersson 2021). For example, migrants are typically considered to be fully integrated in terms of family-demographic behavior when their fertility patterns mirror those of natives. In contrast, immigrant groups with a differing pattern may be considered less integrated, which is often interpreted as an indicator of their social isolation from mainstream society.

In this contribution, we take a life-course, generational and gender perspective to study the fertility transitions of immigrants and their descendants in Sweden. Based on analyses of Swedish register data, we engage with hypotheses and research questions that have been touched upon in much previous family-demographic research on migrants in Europe—but which have previously remained unexplored due to data limitations. A key advantage of register data on immigrants and their descendants in Sweden is that study populations are large enough to allow for the assessment of relationships which in other data sources would appear as products of random variation. With this in mind, the purpose of our study is to highlight the many heterogeneities in the life-course dynamics of immigrants and their descendants in a key European migration context, and to improve our understanding of the mechanisms of their integration.

## Contribution

Our study addresses well-established limitations of previous research on patterns of childbearing among immigrants and their descendants. In particular, our study contributes to the following three aspects:

First, we investigate fertility outcomes across the full range of migrant generations, including immigrants and their descendants. To date, fertility research has overwhelmingly focused on immigrants—leaving their descendants understudied. This research imbalance has arisen from data constraints, the youthful nature of the second generation, and is especially pronounced within the European context (Kulu et al. 2017). In addition, research has often failed to account for the heterogeneity of the second generation and typically does not distinguish whether individuals have two or one foreign-born parent. In our study, we refer to the former group as Generation 2.0 and the latter as Generation 2.5. So far, little is known about the impact of endogamous and exogamous unions on the fertility patterns of the children born in those unions. In our study, we further distinguish whether the mother or the father is an immigrant for the descendants with one foreign-born parent. For immigrants, we distinguished between individuals who moved as adults, and those who moved during their childhood. The latter are both: descendants of immigrants and immigrants themselves. We refer to this group as Generation 1.5. By including this specification, we can assess whether spending parts of the childhood in Sweden, makes this group more similar to either Swedish-born individuals or to immigrants who did not grow up in Sweden.

Second, we investigate the extent to which the Swedish context may impact the childbearing of immigrants and their descendants by studying selected groups from contexts with very different fertility regimes. We provide in-depth information on migrants and their descendants with a background in Poland, Southern Europe, India, Turkey, North Africa, and the other Nordic countries (Denmark, Finland, Iceland, and Norway). The first two backgrounds are typically reported as low-fertility contexts (Mussino, Wilson and Andersson 2021). In contrast, the next three backgrounds have typically represented high-fertility backgrounds in studies of migrant fertility (Andersson 2004, Milewski 2007, Robards and Berrington 2016). Migrants from other Nordic countries stem from contexts that are very similar to Sweden, but individuals differ from natives Swedes by their migration experience (Andersson 2004). Theoretical models on fertility suggest that individuals from low-fertility backgrounds may display increasing fertility levels for those generations with more extended exposure to the Swedish context (Tønnesen and Mussino 2019, Mussino, Wilson and Andersson 2021). In contrast, the opposite tendency would hold for migrants and their descents from high-fertility backgrounds (Dubuc 2012).

The third contribution arises from studying childbearing separately for men and women. This represents an emerging field of research as much previous research on migrant fertility has tended to focus on women only. However, the timing of migration and family formation may be interlinked in very different ways for migrant men and women. A gendered approach is thus essential when exploring whether immigrant men and women adjust differently to the Swedish context.

## The Swedish Context: Fertility and International Migration

Sweden's fertility regime has been characterized as “highest-low” (Andersson 2008). This means that fertility levels are typically below the replacement level, but still higher compared to most other Western countries. Sweden's relatively high fertility has often been linked with its progressive and universalistic welfare regime (Neyer and Andersson 2008). Typically, policies in Sweden aim to ensure that labor-market participation is conducive to childbearing. For example, in Sweden, childbearing is supported through a system of subsidized childcare, individual-based taxation, income-replacement parental leave and policies which promote gender equality (Andersson 2008).

Sweden has also been known for its liberal immigration policies and its embracement of multiculturalism. Following the Second World War, Sweden became a distinct country of immigration. During the 1950s and 1960s, it received large numbers of labor immigrants from Finland and other countries to supply Sweden's growing industry. In addition to labor migration, Sweden has attracted many refugees fleeing war and unrest. For example, during the 1950s and 1960s, Sweden welcomed refugees from Eastern Europe, during the 1970s, from Latin America, during the 1980s from Iran, during the 1990s from the former Yugoslavia, during the 2000s, from Iraq, and more recently, since 2015, from Syria.

Sweden's multicultural policies can be traced back to the 1960s and 1970s (Borevi 2013). In the 1960s, Sweden extended its social welfare system to its immigrant population. The change ensured that immigrants had the same social rights as the rest of the population. The policy reflected concerns that immigrants would otherwise contribute to a more stratified society. The move to universal social rights sought to ensure a more socially cohesive society. In 1975, the Swedish government adopted a new immigration policy which sought to bring “equality, freedom of choice and partnership” to immigrants (Borevi 2013). Under the new policies, immigrants would be encouraged to maintain their cultural distinctiveness whilst simultaneously be granted equal rights to participate in Swedish society.

Turkish migration to Sweden began already in the 1960s with the arrival of work-seeking men who were later followed by their wives and families. Qualitative research by Bayram et al. (2009) hints at a strong attachment of first- and second-generation Turks in Sweden to Turkish culture and identity, alongside a social distance to Swedish natives. Many study participants did not support intermarriage with a Swedish native and favored socialization with fellow Turks. However, immigrants from Turkey stem from several different cultural belongings: Kurdish, Turkish, and Syriac/Assyrian.

In contrast to Turkish migration, immigration from India is a relatively new development with levels increasing threefold since the turn of the new century (SCB 2016). Given that this is a new immigrant group, Myrvold (2012) has recommended that researchers should pay more attention to the integration patterns of this population. Myrvold (2012) shows that Indian immigrants are relatively well educated and have strong presence in the IT and healthcare sectors.

Immigrants from Northern Africa come from several countries and display a significant degree of cultural diversity. They stem from different Arabic and Berber speaking areas of the region.

In the late 1940s and 1950s, migration from Poland was heavily restricted by policies of the communist regime. However, following the easements of international travel in the late 1960s and 1970s, increasing numbers of Poles acquired passports and relocated to Sweden permanently. Still, it was not until the collapse of the Soviet Union in the early 1990s when Poles could freely migrate, a pattern which was further extended through Poland's accession to the European Union in 2004. Today, immigrants from Poland constitute the fourth largest immigrant group in Sweden (Lindström, Mussino and Olah 2022). During most of the time, female Polish migrants have outnumbered those of males, and many have married Swedish men. Polish migrants in Sweden have relatively high levels of education, and many have skills that are easily transferable to the Swedish labor market (Józefowicz 1996).

International migrants from Southern Europe come from several countries and thus make for a less cohesive group in Sweden. However, they all come from countries with strongly familialistic systems and fertility regimes that are characterized by low levels of childbearing (Mussino, Wilson and Andersson 2021). As Polish migrants, migrants from Southern Europe tend to have high levels of education and a substantial number of transferable skills when entering the Swedish labor market.

## Theoretical Considerations and Previous Research

### Explaining Immigrant Fertility

The body of literature on immigrant fertility in Europe has grown rapidly (for reviews, see: Kulu et al. 2019; Andersson 2021). Four hypotheses have been highlighted to explain childbearing among immigrants. Each hypothesis approaches immigrant fertility from a distinct angle. Despite their distinct angle, the hypotheses are not mutually exclusive, and researchers often argue that multiple hypotheses may simultaneously be supported and that they are often interrelated (e.g., Lindström, Mussino and Olah 2022).

The *socialization hypothesis* argues that the fertility behavior of immigrants mainly reflects the fertility preferences that prevailed in their childhood origin (Cygan-Rehm 2014). Socialization can be considered a lifelong process, which can be divided into different stages, such as primary and secondary socialization. Primary socialization takes place during childhood with behavioral traits transmitted from family, school, and the wider community (Kulu et al. 2019; Andersson 2021). Secondary socialization occurs in adulthood whenever an individual encounters a new environment or context such as moving to a new country.

The *adaptation hypothesis* proclaims that immigrants will adapt to the social, cultural, and economic situation in the host country (Cygan-Rehm 2014). As migrants are exposed to a new context, their fertility levels will converge with that of natives. Proponents of the adaptation hypothesis argue that convergence in fertility behavior may take place rather rapidly after moving to a new country. The adaptation in behavior does not necessarily reflect a process of acculturation but rather that immigrants are exposed to a new socioeconomic and institutional setting with its’ constraints and incentives in relation to family formation (Andersson 2008, 2021).

The *selection hypothesis* builds upon the observation that immigrants are a distinctive group when compared to both, nonmovers in their country of origin and natives in their destination country (Hervitz 1985; Kulu 2005; Blau and Kahn 2007; Lindström, Mussino and Olah 2022). Research has shown that migrants typically arrive at young ages and tend to be positively selected in terms of education and health. In addition to human capital, migrants may possess distinct personality traits including being adventurous, taking risks, and being ambitious (Massey et al. 1993). Aspects of selection among immigrants add a further layer of complexity. For example, the positive selection of immigrants by educational attainment can lead to relatively low fertility of migrants from high-fertility countries.

The *hypothesis of interrelated life events* predicts that immigrants, on average, experience elevated fertility shortly after a migration event. The reason for high fertility is that migration, marriage, and childbearing are often interrelated in individuals’ lives (Milewski 2007) and that it is more common to move to a new country before becoming a parent than realizing these life course events in the opposite order. Many women also move from one country to another to marry or to join a partner and will have a child soon after migration (Andersson 2004; Kulu 2005).

Previous research on the fertility of immigrants to Sweden provides support for several of the hypotheses presented above. Studies show that the hypotheses are not mutually exclusive and may have different explanatory power at different stages of migrants’ lives. For example, Andersson (2004) demonstrates strongly elevated fertility rates of newly arrived immigrant women to Sweden, which suggests that family formation and migration are often highly interlinked events in the life of individuals. The same study also shows a process of rapid adaptation in terms of fertility rates with increasing duration of residence in Sweden. Andersson and Scott (2005, 2007) provide further insight into these patterns of adaptation and how they extend to the relationships between socioeconomic and labor market characteristics and fertility outcomes. Mussino, Wilson and Andersson (2021) show that the upward fertility adaptation of immigrants from low-fertility contexts appear less strong than adaptation in the other direction. The study also extends the focus to cover the childbearing outcomes of children who arrived in Sweden during childhood and differences in fertility outcomes by their different ages of migration to this destination.

### Fertility of the Descendants of Immigrants

In contrast to the many studies on immigrants, childbearing among the second generation is less explored. While fertility patterns of the first generation are strongly influenced by the country of origin, the second generation is primarily exposed to a different environment. For example, many descendants of immigrants grow up under the influence of the majority population and may adopt or assimilate seamlessly into cultural and social norms of the mainstream society (Kulu et al. 2019). Hence, the *assimilation hypothesis* predicts that fertility behavior of the descendants is similar to that of the majority population.

Second-generation individuals are likely to be influenced by their immigrant parents. In addition, a wider immigrant community may matter, and some descendants may socialize into a minority subculture. *The subculture hypothesis* predicts that the descendants of immigrants exhibit specific childbearing patterns that are different from those of the majority population—assuming that the immigrant population differs from the native population (Kulu et al. 2019).

Both hypotheses provide a valuable framework for approaching the population subgroups which are located in between the first and second generation, or the second generation and the native population. For example, some immigrants move with their children. As these children were socialized in at least two different contexts, they are located in between immigrants who moved as adults and the descendants of immigrants. This group, typically referred to as Generation 1.5, may thus exhibit fertility patterns that are similar to both, immigrants and their descendants. Another example are individuals who are descendants of one immigrant and one nonmigrant: Generation 2.5. It can be assumed that childbearing patterns among Generation 2.5 are very similar to those of the native population. Mainly due to data constraints, previous research usually aggregates Generation 2.0 and 2.5. However, it has been argued that analyses which treat Generation 2.5 as a distinct group from natives, as well as distinct from Generation 2.0, provide more solid insight into the integration patterns of descendants of immigrants (Karthick Ramakrishnan 2004). While the role of exogamous relationships for assimilation processes has received more attention recently (Braack and Milewski 2019), still, very little is known about the fertility patterns of Generation 2.5.

Research on the fertility of the descendants of immigrants has been more advanced in Sweden than in other parts of Europe. Scott and Stanfors (2011) found that descendants of immigrants generally had lower fertility than Swedish natives. In a comparative study of fertility among the descendants of immigrants across six European countries, Kulu et al. (2017) found that the variation between second-generation groups was the smallest in Sweden. The authors attribute this to the equalizing effect of Sweden's welfare system, which has enabled ethnic minorities to integrate better than in many other societies. Finally, Andersson, Persson and Obućina (2017) found that most groups of descendants of immigrants had lower fertility than Swedish natives, and that this was exhibited by lower rates of first and second births.

## Expectations

Based on previous research and our study design, we expect to find the following:

First, most immigrant groups are expected to exhibit elevated fertility levels shortly after arrival in Sweden (Andersson 2004). However, it is likely that we observe such elevated fertility mostly for women and less so for men who may have arrived in Sweden before being joined by their partner. We aim at determining the extent to which such gender differences in behavior differ between migrants from different countries of origin.

Second, all migrant groups are expected to experience some degree of fertility change with increasing duration of residence in Sweden. These patterns may differ between immigrants from high- and low-fertility contexts. As hypothesized in previous research for Sweden, we assume the adaption from high-fertility backgrounds to the Swedish levels to occur faster than the adaption from low-fertility background (Mussino, Wilson and Andersson 2021).

Third, we expect the descendants of immigrants to generally exhibit childbearing patterns that are relatively similar to those of the native Swedish population (Kulu et al. 2017). However, it is less clear whether those who arrived in Sweden as children are more similar in behavior to either adult immigrants or to descendants of immigrants who were born in Sweden (Mussino, Wilson and Andersson 2021).

Fourth, descendants of immigrants with only one migrant parent are expected to display fertility behavior that is closer to that of natives than for the descendants of two immigrant parents. In the case of the descendants of one immigrant and one native-born parent, we also aim to determine whether fertility levels differ with respect to whether it was the mother or the father who was a first-generation immigrant—or a native Swede.

## Data and Methods

### Data

For this study, we used individual-level register data from Sweden. These routinely collected, administrative data cover the entire population with legal residence in Sweden. The quality of Swedish register data, especially its completeness and accuracy, is widely acknowledged (Ludvigsson et al. 2016).

We had access to longitudinal data from the Swedish total population register (TPR) covering the period 1968–2017. The TPR covers all major demographic events, such as childbirths, marriages, deaths, or international migrations of the Swedish population. All individuals covered in the TPR receive a unique personal identification number, which is assigned either immediately after being born in Sweden or after having registered in Sweden after arrival (Ludvigsson et al. 2009). With an anonymized version of this identification number, we followed individuals throughout different data sets via deterministic record linkages. For this study, we linked records from the TPR with data from the longitudinal integrated database for health insurance and labor market studies—the LISA database. The purpose of these linkages is to provide controls for socioeconomic characteristics that are related to parity-specific fertility, which may obscure the role of migration background if not properly considered. The set of variables is quite conventional, but the exact operationalization is determined by the structure of available data.

Data from the LISA database are available since 1990 and cover all individuals aged 16 and older who are registered in Sweden (Ludvigsson et al. 2019). Data are updated by Statistics Sweden on an annual basis and information typically reflect entire calendar years (SCB 2019). We had access to data covering the period between 1990 and 2016. From LISA, we obtained information on individuals’ education, any unemployment benefits and student allowances, as well as whether individuals were in employment. The indicators reflecting unemployment benefits, student allowances, and employment status were binary indicators capturing the situation throughout the entire respective calendar year. Given this structure of the data, it is possible that records indicate that individuals have received unemployment benefits (due to a spell of unemployment during the year) while also having experienced employment during the same year. Our indicator for educational attainment is based on a harmonized classification of Swedish educational attainment codes, captured by the *Svensk utbildningsnomenklatur* (SUN) system). This classification allowed us to define some broad levels of educational levels—primary, secondary, and tertiary—and to capture these levels consistently over time, focusing on the highest level of education completed (SCB 2019).

### Reconstructing Birth Histories and Population Subgroups

Swedish register data provide the opportunity for intergenerational linkages as the personal identification number of children and parents can be linked (Wall-Wieler et al. 2018). These intergenerational linkages allowed us to reconstruct detailed birth histories for all individuals, providing an exact date of birth for each parity progression. Furthermore, it enabled us to capture the parents’ country of origin and to establish the migration history of individuals’ parents. All first- and second-generation groups were further differentiated into different country-of-origin backgrounds, capturing a wide range of fertility-relevant backgrounds.

We split immigrants into Generation 1.0 and Generation 1.5, reflecting the age at which an individual arrived in Sweden. Generation 1.0 is defined as immigrants who arrived in Sweden at the age of 16 or above. Meanwhile, Generation 1.5 reflects individuals who arrived in Sweden at age 15 or younger.

We captured descendants of immigrants as Generation 2.0 and Generation 2.5. This differentiation indicates whether an immigrant's descendant was an offspring from an endogamous or exogamous relationship. Generation 2.0 encompasses second-generation offspring whose both parents were born outside of Sweden, with both parents having the same country-of-origin background—and thus were in an endogamous relationship. Generation 2.5 corresponds to descendants of immigrants whose parents were in an exogamous relationship with a native Swede. This means that one parent was born in Sweden while the other parent was born outside of Sweden. For all individuals of Generation 2.5, we also distinguished whether it was the mother or the father who was a first-generation immigrant.

### Study Population and Study Period

In the Swedish TPR, we identified all individuals born between 1941 and 2000, and who had plausible information on sex, year of birth, and country of birth, as well as plausible information on their parents’ demographic background (*N* = 8,080,338). Overall, the amount of implausible information was very small, underlining the high quality of the data. We chose this range of birth cohorts as we focused on the age range 15–49 throughout the study period, which lasted from January 1, 1991, to December 31, 2017.

In a next step, we excluded all individuals for which the coverage in the register was less than 30 consecutive days and who did not reside in Sweden when aged 15–49 during the study period. Major causes of exclusion were death or outmigration before age 15 or immigration after age 50. This reduced the size of our study population to 7,286,140 individuals.

We then accounted for overcoverage in the Swedish register. Overcoverage represents a common phenomenon in most administrative data sources, including the Swedish registers (Monti et al. 2020). A major factor contributing to overcoverage in population registers is the underreporting of outmigration, particularly among young adults. For this purpose, we utilized our own register-trace algorithm. Using this algorithm, we identified periods of inactivity among studied individuals based on missing information regarding income, unemployment benefits, student allowances, labor market activity, and educational attainment. Identifying and correcting for periods of inactivity reduced the size of the study population to 7,265,899 individuals.

Of the remaining individuals, we identified all women and men who were at risk of a first birth in Sweden. This meant that we excluded all individuals who had already had their first child before the study period began. This reduced the final size of the study population to 5,322,242 individuals.

Similarly, for analyses of higher parity births, we followed mothers and fathers for a second (or third) birth, for which we had recorded a first (and second) birth during the study period in Sweden. Individuals experiencing a multiple birth were captured, but excluded from further analyses of higher parity births.

For Swedish-born individuals and foreign-born individuals who arrived in Sweden at ages 15 or less, the observation period started on January 1, 1991, or the date the individual turned 15, whichever occurred last. The period of observation for migrants who arrived in Sweden after having turned 16 began on January 1, 1991, or the date of earliest arrival in Sweden—whichever occurred last. The end of each individual's observation period was defined as the earliest of the following events: first outmigration, no sign of activity in the register, death, reaching age 50, December 31, 2017, or having a third child.

### Data Setup

Based on the start and end dates of individuals’ observation periods, we created series of one-year episodes for all women and men, and incorporated our socioeconomic covariates in a time-varying manner.

To minimize problems of reversed causality, we incorporated all socioeconomic covariates with a one-year lag. This means that all socioeconomic data for a parity-specific birth reflect the circumstances of the year the child was conceived—rather than the circumstances the child was born. In a small number of cases, we corrected for delays in the recording of information on education for foreign-born individuals, which are often updated in the early years after arrival in Sweden (Saarela and Weber 2017).

Our statistical modeling was based on parity-specific data where each data subset captured only the relevant at-risk population for the respective parity progression, reflecting the start and end dates of each relevant observation spell. For the first birth analyses, we used age as the time scale. For the second- and third-birth analyses, we used time since previous birth as the time scale.

### Statistical Modeling

We estimated Kaplan–Meier survival curves for the transitions to a first, second, and third child (Kaplan and Meir 1958). Immigrants entering Sweden after age 15 represent late entries in the first birth analyses as we used age as the time scale. We therefore present no survival curves for immigrants with respect to first births, as they would not be comparable to those of the other population subgroups.

We used Cox proportional hazards models to study differences in first, second, and third birth rates between population subgroups (Cox 1972). The approach provides information on the intensity of childbearing in relation to a suitable reference category, but not always on the quantum of fertility: for the latter information we have to rely on our descriptive information from the Kaplan–Meier survival curves. All models were estimated separately for men and women. In all models, native Swedish men and women were the reference category. Cox models for first childbearing included the following time-varying covariates: period (calendar year), level of education, student allowances, unemployment benefits, employment status, as well as time since first arrival for immigrants who arrived in Sweden as adults. Models for second and third birth included the following time-varying covariates: period (calendar year) level of education, student allowances, unemployment benefits, employment status, and age at last previous birth. The socioeconomic variables are mainly included as controls while the presentation of results focuses on the role of migration background in relation to parity-specific fertility. All full models with results also for the socioeconomic variables are presented in supplemental tables.

We report hazard ratios (HRs) and corresponding 95% confidence intervals (95% CIs). Data preparation and statistical modeling were carried out using R, Version 4.1.1 (R Core Team 2021). Data preparation was carried out using the R-dialect “data.table” (Dowle et al. 2021). The R package “survival” was used for time-to-event analyses (Therneau et al. 2021).

Access to the data is managed by Stockholm University. The data can be accessed for research under ethical approval from the regional ethics board in Stockholm, Sweden (Project: FORTE (Forskningsrådet om Hälsa, Arbetsliv och Välfärd) for a project on Migrant Trajectories, grant number 2016-07105). No additional ethical approval was required for data access, data linkages, and the results of data analysis presented in this study.

## Results

### First Birth

We studied 5,322,242 men and women who were at risk of having a first child in Sweden between 1991 and 2017. Within this period, we observed 2,315,687 first births. An overview of the population at risk of becoming a parent is provided in Table 1. Men and women with a native Swedish background formed the largest population subgroup (65.10%), followed by first-generation immigrants (Generation 1.0: 17.71%; Generation 1.5: 5.73%), and the second generation (Generation 2.0: 4.28%; Generation 2.5M: 3.47%; Generation 2.5F: 3.71%). One notable feature is that a majority of those who in many studies are labeled as the second generation are part of the generation that we have defined as 2.5. A more detailed overview of the population at risk by country-of-origin background is provided in Table S1.

**Table 1.** Overview of the Study Population at Risk of First Birth and Number of First Births by Aggregated Population Subgroups.

Population subgroup	*N* Males	*N* Females	*N* Total	*N* Total (%)	Males birth	Females birth
Native Swedish	1,858,844	1,605,907	3,464,751	65.10	815,448	827,415
Generation 1.0	534,847	407,913	942,760	17.71	152,847	161,478
Generation 1.5	159,601	145,297	304,898	5.73	53,122	60,687
Generation 2.0	119,158	108,639	227,797	4.28	37,401	41,018
Generation 2.5—Mother Migrant	98,929	85,638	184,567	3.47	39,370	40,070
Generation 2.5—Father Migrant	103,216	94,253	197,469	3.71	41,707	45,124

**Figure 1.**
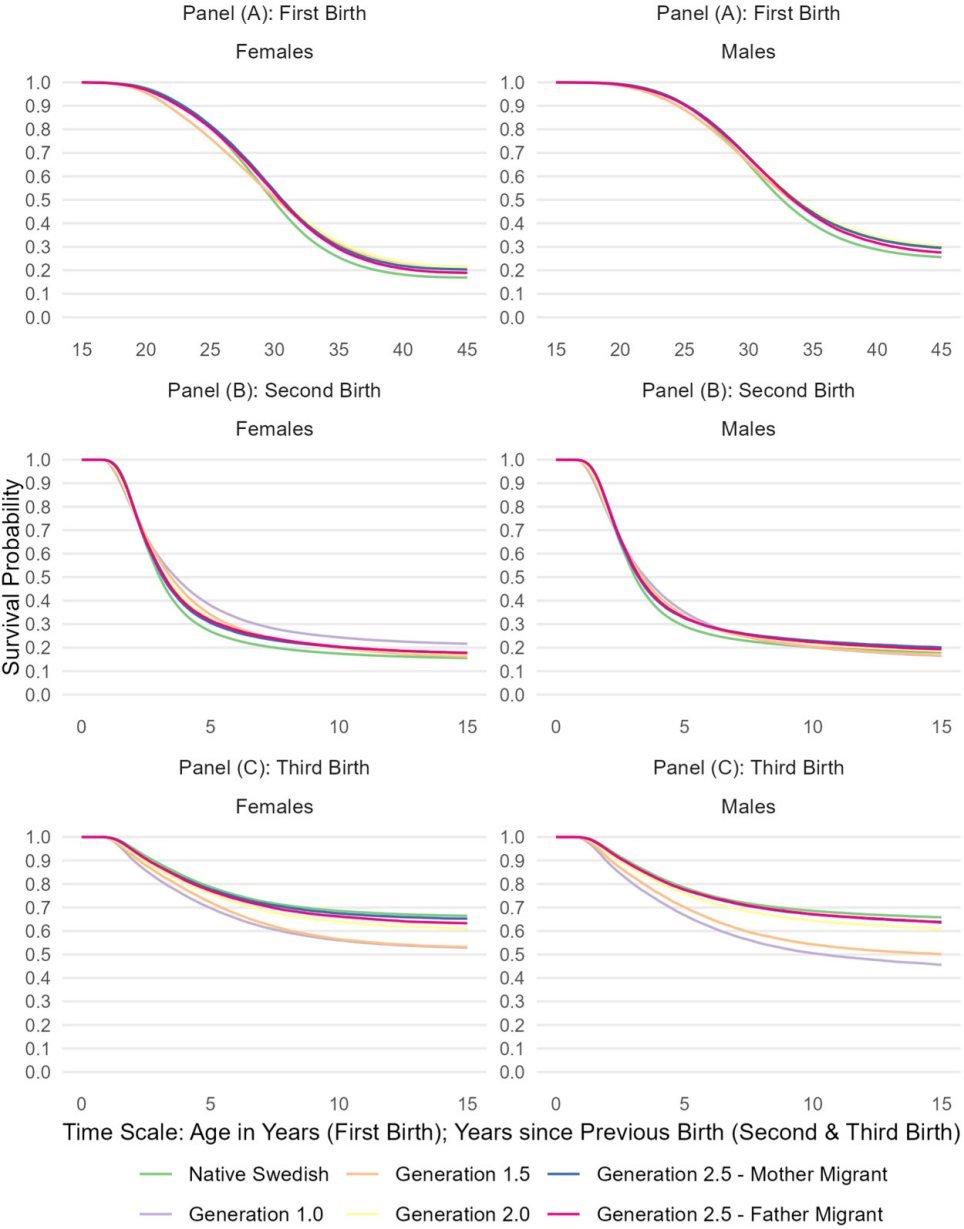
Kaplan–Meier Survival Curves for Transitions to Any First, Second, and Third Birth.

*Note:* The x-axis is truncated in the plot and does not correspond to the upper age limit (age 49) when censoring.

Figure 1 Panel A shows Kaplan–Meier survival curves for the transition to a first birth. The aggregated population subgroups follow a very similar trajectory of transitions, with women becoming a parent at somewhat younger ages than men. For example, 19.0% (95% CI: 18.9%–19.1%) of native Swedish women had become a mother already at age 25 while the corresponding level of fathers was 9.4% (9.3%–9.5%). Men and women of Generation 1.5 had a first birth slightly earlier than their counterparts from the other population subgroups. For example, 23.8% (23.5%–24.0%) of women of Generation 1.5 had become a mother at age 25—in comparison to 19.1% (18.8%–19.4%) of the women in Generation 2.0. In contrast, after age 25, native Swedish men and women tended to become parents at higher rates than individuals with a migration background.

As shown in Figure 1, the levels of ultimate childlessness are lower among women than among men. For example, 16.9% (95% CI: 16.8%–17.0%) of native Swedish women were childless at age 45 while the corresponding level of childless men was 25.6% (25.5%–25.7%). Overall, the levels of ultimate childlessness were consistently smallest among native Swedish women and men when compared to the subgroups with a migration background. For example, 21.5% (21.2%–21.9%) of women of Generation 1.5 as well as 21.6% (21.2%–22.0%) of women of Generation 2.0 were childless at age 45.

The levels of childlessness in our synthetic cohorts tend to be higher than what has been observed for the completed fertility of actual birth cohorts in Sweden (Jalovaara et al. 2019). This difference is likely due to the fact that first-birth rates in Sweden declined during several parts of the study period on which we base the estimations in our present study (Ohlsson-Wijk and Andersson 2022).

Next, we examine differences in first birth rates across population subgroups based on multivariate Cox proportional hazards models. As shown in Table 2, first birth rates were elevated among newly arrived immigrants in Sweden, in comparison to native Swedes. For example, for women from Turkey, first birth rates were substantially increased in the first two years after arrival in Sweden when compared to native Swedish women (HR: 5.62 (5.43–5.81)). A similar pattern holds for the other groups of first-generation women, except for those arriving from Southern Europe. Interestingly, a similar pattern also holds for most groups of newly arrived men, with immigrants from Turkey again standing out with distinctly elevated first-birth rates, in comparison to native Swedes, in their first two years in Sweden (HR: 3.93 (3.79–4.08)). In contrast, at longer durations since migration to Sweden, first-birth rates were generally lower, when compared to native Swedes. However, for adult immigrants from Turkey they remained higher than among native Swedish women and men also at more extended stays in the new country (HR: 1.37 (1.29–1.46) and 1.78 (1.71–1.86)). We found a similar pattern to hold for adult immigrants from North Africa. In contrast, we found a specific pattern of depressed fertility, in comparison to native Swedes, among immigrants from the low-fertility context of Southern Europe. This was most pronounced for women, but we also found that their first birth rates increased somewhat over time in Sweden (HR: 0.63 (0.59–0.68), 0.84 (0.79–0.89), and 0.89 (0.83–0.95)). For Generation 1.0, we thus found some patterns of polarization by high- and low-fertility backgrounds but more clearly, a pattern of distinct changes in behavior over time since migration to Sweden, which were relatively consistent among women as well as men.

**Table 2.** Results of Cox Proportional Hazards Models for Transitions to First Birth by Population Subgroup and Country-of-Origin Background, Separately for Men and Women.

Population subgroup	Females HR	(95% CI)	Males HR	(95% CI)
Native Swedish (Reference)				
Generation 1.0—Nordic (0,2]	1.08	(1.05–1.11)	1.63	(1.57–1.68)
Generation 1.0—Nordic (2,5]	1.17	(1.14–1.21)	1.40	(1.35–1.45)
Generation 1.0—Nordic (5,Inf]	0.86	(0.83–0.88)	0.84	(0.82–0.87)
Generation 1.0—Poland (0,2]	1.84	(1.78–1.90)	1.18	(1.12–1.23)
Generation 1.0—Poland (2,5]	1.36	(1.30–1.41)	1.22	(1.16–1.27)
Generation 1.0—Poland (5,Inf]	0.95	(0.91–0.99)	1.02	(0.97–1.07)
Generation 1.0—Turkey (0,2]	5.62	(5.43–5.81)	3.93	(3.79–4.08)
Generation 1.0—Turkey (2,5]	2.88	(2.73–3.03)	1.88	(1.80–1.97)
Generation 1.0—Turkey (5,Inf]	1.37	(1.29–1.46)	1.78	(1.71–1.86)
Generation 1.0—Europe South (0,2]	0.63	(0.59–0.68)	0.96	(0.90–1.02)
Generation 1.0—Europe South (2,5]	0.84	(0.79–0.89)	0.96	(0.90–1.01)
Generation 1.0—Europe South (5,Inf]	0.89	(0.83–0.95)	0.99	(0.94–1.04)
Generation 1.0—Africa North (0,2]	5.93	(5.73–6.13)	2.37	(2.26–2.48)
Generation 1.0—Africa North (2,5]	2.52	(2.36–2.70)	1.11	(1.05–1.17)
Generation 1.0—Africa North (5,Inf]	1.44	(1.31–1.57)	1.77	(1.69–1.85)
Generation 1.0—India (0,2]	1.97	(1.86–2.09)	1.00	(0.93–1.07)
Generation 1.0—India (2,5]	1.69	(1.57–1.82)	0.81	(0.75–0.88)
Generation 1.0—India (5,Inf]	1.12	(1.00–1.26)	1.18	(1.09–1.26)
Generation 1.0—All Other (0,2]	2.48	(2.45–2.50)	1.78	(1.76–1.80)
Generation 1.0—All Other (2,5]	1.56	(1.54–1.58)	1.52	(1.50–1.54)
Generation 1.0—All Other (5,Inf]	1.09	(1.08–1.11)	1.47	(1.46–1.49)
Generation 1.5—Nordic	0.97	(0.95–0.99)	0.91	(0.89–0.93)
Generation 1.5—Poland	0.89	(0.85–0.92)	0.95	(0.91–1.00)
Generation 1.5—Turkey	1.29	(1.24–1.35)	1.58	(1.52–1.64)
Generation 1.5—Europe South	0.76	(0.70–0.82)	0.98	(0.92–1.05)
Generation 1.5—Africa North	1.21	(1.08–1.36)	1.12	(1.00–1.26)
Generation 1.5—India	0.79	(0.76–0.82)	0.68	(0.64–0.72)
Generation 1.5—All Other	1.03	(1.02–1.04)	1.07	(1.06–1.08)
Generation 2.0—Nordic	1.04	(1.02–1.05)	0.97	(0.95–0.98)
Generation 2.0—Poland	0.72	(0.68–0.77)	0.84	(0.79–0.90)
Generation 2.0—Turkey	1.07	(1.03–1.10)	1.11	(1.07–1.15)
Generation 2.0—Europe South	0.73	(0.69–0.78)	0.89	(0.84–0.94)
Generation 2.0—Africa North	0.84	(0.77–0.92)	0.88	(0.80–0.98)
Generation 2.0—India	0.42	(0.35–0.50)	0.49	(0.40–0.61)
Generation 2.0—All Other	0.81	(0.80–0.83)	0.89	(0.88–0.91)
Generation 2.5—Mother Migrant—Nordic	0.96	(0.95–0.97)	0.93	(0.92–0.95)
Generation 2.5—Mother Migrant—Poland	0.76	(0.73–0.80)	0.81	(0.77–0.85)
Generation 2.5—Mother Migrant—Turkey	0.78	(0.60–1.01)	0.84	(0.64–1.11)
Generation 2.5—Mother Migrant—Europe South	0.84	(0.79–0.91)	1.00	(0.93–1.07)
Generation 2.5—Mother Migrant—Africa North	0.68	(0.54–0.86)	0.76	(0.58–0.99)
Generation 2.5—Mother Migrant—India	0.72	(0.60–0.87)	0.86	(0.70–1.04)
Generation 2.5—Mother Migrant—All Other	0.83	(0.82–0.85)	0.91	(0.89–0.93)
Generation 2.5—Father Migrant—Nordic	1.04	(1.03–1.05)	1.01	(0.99–1.02)
Generation 2.5—Father Migrant—Poland	0.87	(0.81–0.94)	0.96	(0.88–1.04)
Generation 2.5—Father Migrant—Turkey	0.88	(0.82–0.96)	0.97	(0.89–1.06)
Generation 2.5—Father Migrant—Europe South	0.88	(0.85–0.91)	0.95	(0.92–0.98)
Generation 2.5—Father Migrant—Africa North	0.86	(0.81–0.91)	0.90	(0.84–0.96)
Generation 2.5—Father Migrant—India	0.76	(0.66–0.88)	0.84	(0.74–0.97)
Generation 2.5—Father Migrant—All Other	0.89	(0.87–0.90)	0.95	(0.93–0.96)

*Note:* All HRs are controlled for period, education, unemployment benefits, student allowances, and employment status. The HRs for first generation immigrants are classified by the duration since their registered immigration to Sweden, with three yearly categories each.

First birth rates among immigrants who arrived in Sweden as children tended to be more clearly defined by the fertility context in their countries of origin and we observe a clear divide into groups with high- and low-fertility behavior. For example, first birth rates were elevated among men who arrived in Sweden as children from Turkey (HR: 1.58 (1.52–1.64)) but depressed among women who arrived as children from Southern Europe (HR: 0.76 (0.70–0.82))—when compared with the first birth rates observed among native Swedes.

For most studied second-generation groups, we found that first birth rates were generally lower than the levels observed among native Swedish women and men. While there was still some reflection of high- and low-fertility backgrounds, it was only the descendants of Turkish immigrants that had slightly higher first birth rates than native-Swedish women and men (HR: 1.07 (1.03–1.10) and 1.11 (1.07–1.15)).

For both women and men, we found that first birth rates among the descendants of immigrants of an exogamous relationship with one foreign-born and one Swedish-born parent were also lower than for native-born Swedish women and men. This holds in particular for the Generation 2.5 group where the mother was the immigrant and the father Swedish-born. If the father was an immigrant and the mother Swedish-born, first-birth rates were generally closer to those of the native-born population.

Our results for first births indicate that there was no clear polarization into high- and low-fertility backgrounds among the descendants of immigrants who are offspring of an exogamous relationship. In this regard, first birth rates among all studied Generation 2.5 groups were depressed but still rather similar to the rates observed among native Swedish women and men. We found a slightly stronger similarity of Generation 2.5 with the native Swedish population among men than among women, and in cases where the mother was the Swedish-born counterpart in the exogamous relationship.

A comprehensive overview of all discussed Cox models, including all utilized covariates and their respective parameter estimates, is provided in Tables S2A and S2B. An overview of the sociodemographic characteristics of the study populations at the time of first birth is provided in Table S3.

### Second Birth

We followed all individuals for which we recorded a first singleton birth in Sweden within the study period for the transition to any second birth. Among the 2,280,584 individuals who were at risk of a second birth, we observed 1,594,838 births within the study period. An overview of the populations at risk of having a second child is provided in Table 3, while a more detailed overview by country-of-origin backgrounds is provided in Table S4.

**Table 3.** Overview of the Study Population at Risk of Second Birth and Number of Second Births by Aggregated Population Subgroups.

Population subgroup	*N* Males	*N* Females	*N* Total	*N* Total (%)	Males birth	Females birth
Native Swedish	802,715	814,752	1,617,467	70.92	571,456	597,714
Generation 1.0	150,740	159,159	309,899	13.59	92,219	93,152
Generation 1.5	52,400	59,865	112,265	4.92	34,560	39,931
Generation 2.0	36,898	40,395	77,293	3.39	24,923	27,446
Generation 2.5—Mother Migrant	38,742	39,448	78,190	3.43	26,651	27,771
Generation 2.5—Father Migrant	41,068	44,402	85,470	3.75	28,152	30,863

As shown by the Kaplan–Meier survival curves in Figure 1 Panels B, and as observed for first births, the aggregated population subgroups followed a rather similar pattern of transition to second birth. There was only a small amount of heterogeneity between the aggregated subgroups, as well as between fathers and mothers. Overall, native Swedish women were the most likely to transition to a second child with 84.4% (84.3%–84.5%) having had a second child within 15 years since becoming a mother. Immigrant women who arrived in Sweden as adults accounted for the largest deviation as somewhat fewer in this group of mothers had a second child (83.7%; 83.3%–84.1%). The differences among groups of fathers were even smaller than among those of mothers.

We used Cox proportional hazards models to study differences in second birth rates, conditional on having experienced a first birth, across population subgroups differentiated by their country-of-origin backgrounds. As shown in Table 4, we observe some heterogeneity between population subgroups also in their second birth behavior.

**Table 4.** Results of Cox Proportional Hazards Models for Transitions to Second Birth by Population Subgroup and Country-of-Origin Background, Separately for Men and Women.

Population subgroup	Females HR	(95% CI)	Males HR	(95% CI)
Native Swedish (Reference)				
Generation 1.0—Nordic	0.93	(0.90–0.95)	0.95	(0.93–0.98)
Generation 1.0—Poland	0.53	(0.52–0.55)	0.59	(0.57–0.62)
Generation 1.0—Turkey	0.99	(0.96–1.02)	1.00	(0.97–1.03)
Generation 1.0—Europe South	0.86	(0.82–0.91)	0.96	(0.92–1.00)
Generation 1.0—Africa North	1.27	(1.22–1.31)	1.13	(1.09–1.17)
Generation 1.0—India	0.62	(0.58–0.66)	0.61	(0.57–0.66)
Generation 1.0—All Other	0.92	(0.91–0.92)	1.09	(1.08–1.09)
Generation 1.5—Nordic	0.84	(0.82–0.87)	0.89	(0.87–0.91)
Generation 1.5—Poland	0.76	(0.72–0.80)	0.77	(0.73–0.81)
Generation 1.5—Turkey	0.99	(0.95–1.04)	1.19	(1.14–1.25)
Generation 1.5—Europe South	0.90	(0.82–0.99)	1.03	(0.95–1.12)
Generation 1.5—Africa North	0.93	(0.81–1.07)	1.10	(0.95–1.27)
Generation 1.5—India	0.78	(0.75–0.82)	0.90	(0.84–0.98)
Generation 1.5—All Other	0.88	(0.87–0.89)	0.99	(0.98–1.00)
Generation 2.0—Nordic	0.91	(0.89–0.92)	0.90	(0.88–0.92)
Generation 2.0—Poland	0.83	(0.77–0.90)	0.92	(0.84–1.00)
Generation 2.0—Turkey	0.96	(0.92–1.00)	1.21	(1.16–1.27)
Generation 2.0—Europe South	0.92	(0.86–0.99)	1.05	(0.98–1.12)
Generation 2.0—Africa North	0.97	(0.87–1.10)	1.11	(0.97–1.27)
Generation 2.0—India	0.99	(0.78–1.26)	1.16	(0.88–1.53)
Generation 2.0—All Other	0.93	(0.92–0.95)	0.98	(0.96–1.00)
Generation 2.5—Mother Migrant—Nordic	0.96	(0.94–0.97)	0.94	(0.92–0.95)
Generation 2.5—Mother Migrant—Poland	0.90	(0.85–0.95)	0.91	(0.85–0.97)
Generation 2.5—Mother Migrant—Turkey	0.76	(0.52–1.10)	0.78	(0.53–1.13)
Generation 2.5—Mother Migrant—Europe South	0.92	(0.84–1.00)	0.88	(0.80–0.96)
Generation 2.5—Mother Migrant—Africa North	0.84	(0.61–1.15)	1.33	(0.93–1.91)
Generation 2.5—Mother Migrant—India	1.12	(0.87–1.42)	0.90	(0.71–1.16)
Generation 2.5—Mother Migrant—All Other	0.97	(0.94–0.99)	0.96	(0.94–0.98)
Generation 2.5—Father Migrant—Nordic	0.93	(0.91–0.94)	0.92	(0.91–0.94)
Generation 2.5—Father Migrant—Poland	0.93	(0.84–1.02)	0.91	(0.82–1.00)
Generation 2.5—Father Migrant—Turkey	0.89	(0.80–0.98)	0.83	(0.74–0.93)
Generation 2.5—Father Migrant—Europe South	0.92	(0.88–0.96)	0.90	(0.86–0.94)
Generation 2.5—Father Migrant—Africa North	0.93	(0.86–1.00)	0.94	(0.87–1.02)
Generation 2.5—Father Migrant—India	1.01	(0.85–1.19)	1.07	(0.91–1.26)
Generation 2.5—Father Migrant—All Other	0.94	(0.92–0.95)	0.96	(0.95–0.98)

*Note:* All HRs are controlled for period, age at previous birth, education, unemployment benefits, student allowances, and employment status.

In particular, most groups of immigrants and descendants of immigrants displayed second-birth rates that were somewhat or markedly lower than those for native Swedes. Poland stands out as a migration origin that is associated with particularly low second birth rates, contextualized against birth rates among native Swedes. This holds for adult immigrant Polish women as well as men (HR: 0.53 (0.52–0.55) and 0.59 (0.57–0.62)). The negative association is also strong for childhood immigrants from Poland but is somewhat attenuated in the second generation, particularly for women and men from exogamous unions with a Swedish-born parent. For immigrants from high-fertility backgrounds, we found that second birth rates were relatively close to the levels observed among the native Swedish population. For example, second birth rates among immigrant women and men from Turkey who arrived in Sweden as adults were not significantly different from the rates observed among native Swedes (HR: 0.99 (0.96–1.02) and 1.00 (0.97–1.03)). The only group with higher second birth rates than for natives Swedes were adult immigrant women and men from North Africa (HR: 1.27 (1.22–1.31) and 1.13 (1.09–1.17).

The observed patterns indicate that second-rate birth rates are relatively high among native Swedes, as compared to patterns in many other countries. In our study, we found a general depression of second birth rates among most second-generation groups in comparison to native Swedes, but the extent of this depression was less strong than for first birth fertility. To some extent, it is related to immigrants having somewhat less compressed second-birth intervals than native Swedes (Andersson et al. 2006). As already observed for first births, there was no clear polarization into groups with high- and low-fertility backgrounds in the second generation and among the offspring of immigrants from exogamous relationships. Differences between the offspring from endogamous and exogamous relationships were rather small and only observed in a small number of cases, such as men who are descendants of Turkish immigrants (HR: Generation 2.0: 1.21 (1.16–1.27); Generation 2.5M: 0.78 (0.53–1.13); Generation 2.5F: 0.83 (0.74–0.93)).

A comprehensive overview of the discussed Cox models for the transition to a second birth is provided in Tables S5A and S5B. Table S6 provides a descriptive overview of the sociodemographic characteristics of the study populations at the time of any second birth.

### Third Birth

Lastly, we followed all individuals for which we previously recorded a first and second singleton birth in Sweden for the transition to any third birth. Among 1,573,041 individuals at risk, we observed 439,917 third births. An overview of the population at risk is presented in Table 5. A more detailed overview by country-of-origin backgrounds is provided in Table S7.

**Table 5.** Overview of the Study Population at Risk of Third Birth and Number of Third Births by Aggregated Population Subgroups.

Population subgroup	*N* Males	*N* Females	*N* Total	*N* Total (%)	Males birth	Females birth
Native Swedish	563,445	589,435	1,152,880	73.29	150,050	156,699
Generation 1.0	91,070	92,015	183,085	11.64	32,711	30,016
Generation 1.5	34,140	39,442	73,582	4.68	11,622	12,925
Generation 2.0	24,597	27,082	51,679	3.29	7,109	7,898
Generation 2.5—Mother Migrant	26,260	27,361	53,621	3.41	7,276	7,445
Generation 2.5—Father Migrant	27,792	30,402	58,194	3.70	7,634	8,532

Figure 1 Panels C present Kaplan–Meier survival curves for the transition of two-child parents to a third birth. Here, our results indicate a strong polarization in outcomes among the aggregated groups and between the generations in Sweden. This polarization, which we did not observe for first and second births, became clearly visible with respect to the proportions of individuals who have had a third birth within the 15-year period following the second birth. For example, among immigrant two-child fathers who arrived as adults in Sweden, 54.4% (53.9%–54.9%) had a third child within 15 years since the birth of the second child. For native Swedish men, the corresponding proportion was 34.2% (34.0%–34.3%).

Results of the Cox proportional hazards models for the transition to a third birth, conditional on having had a second child, are shown in Table 6. At this point, results indicated quite some heterogeneity among the studied country-of-origin subgroups. For example, we found clear evidence of elevated third birth rates among immigrant groups from previous high-fertility contexts, in comparison to native Swedes. We observed this phenomenon among immigrant women and men who arrived in Sweden as adults as well as those who arrived during childhood. For example, third birth rates among men who arrived from Turkey as an adult were elevated (HR: 1.59 (1.52–1.66)) as well as among those who arrived as children (HR: 1.97 (1.86–2.09)), when compared to native Swedish men. In contrast, third birth rates appeared depressed among adult immigrant women and men from Poland (HR: 0.68 (0.62–0.74) and 0.78 (0.70–0.86)), when contextualized against native Swedes.

**Table 6.** Results of Cox Proportional Hazards Models for Transitions to Third Birth by Population Subgroup and Country-of-Origin Background, Separately for Men and Women.

Population subgroup	Females HR	(95% CI)	Males HR	(95% CI)
Native Swedish (Reference)				
Generation 1.0—Nordic	1.13	(1.08–1.19)	1.05	(0.99–1.11)
Generation 1.0—Poland	0.68	(0.62–0.74)	0.78	(0.70–0.86)
Generation 1.0—Turkey	1.22	(1.16–1.29)	1.59	(1.52–1.66)
Generation 1.0—Europe South	1.00	(0.87–1.14)	1.02	(0.93–1.13)
Generation 1.0—Africa North	1.97	(1.87–2.08)	3.07	(2.92–3.23)
Generation 1.0—India	0.75	(0.64–0.88)	1.11	(0.95–1.29)
Generation 1.0—All Other	1.40	(1.38–1.42)	1.91	(1.88–1.93)
Generation 1.5—Nordic	1.03	(0.98–1.08)	1.07	(1.02–1.12)
Generation 1.5—Poland	0.93	(0.84–1.03)	0.97	(0.87–1.08)
Generation 1.5—Turkey	1.64	(1.54–1.75)	1.97	(1.86–2.09)
Generation 1.5—Europe South	1.00	(0.83–1.21)	1.19	(1.03–1.38)
Generation 1.5—Africa North	1.57	(1.26–1.95)	1.70	(1.36–2.13)
Generation 1.5—India	0.90	(0.81–0.99)	1.00	(0.85–1.17)
Generation 1.5—All Other	1.18	(1.16–1.21)	1.41	(1.38–1.44)
Generation 2.0—Nordic	1.02	(0.99–1.05)	1.04	(1.01–1.08)
Generation 2.0—Poland	0.77	(0.65–0.91)	0.96	(0.80–1.14)
Generation 2.0—Turkey	1.52	(1.42–1.61)	1.63	(1.51–1.75)
Generation 2.0—Europe South	0.88	(0.76–1.02)	1.01	(0.89–1.15)
Generation 2.0—Africa North	1.51	(1.23–1.85)	1.72	(1.34–2.19)
Generation 2.0—India	0.83	(0.45–1.55)	1.52	(0.88–2.62)
Generation 2.0—All Other	1.01	(0.97–1.05)	1.04	(1.00–1.08)
Generation 2.5—Mother Migrant—Nordic	1.04	(1.01–1.06)	1.07	(1.04–1.10)
Generation 2.5—Mother Migrant—Poland	1.07	(0.95–1.20)	1.00	(0.88–1.15)
Generation 2.5—Mother Migrant—Turkey	1.21	(0.61–2.42)	0.56	(0.18–1.75)
Generation 2.5—Mother Migrant—Europe South	0.99	(0.83–1.18)	1.13	(0.95–1.33)
Generation 2.5—Mother Migrant—Africa North	0.85	(0.40–1.78)	0.88	(0.42–1.84)
Generation 2.5—Mother Migrant—India	1.58	(1.00–2.51)	0.79	(0.44–1.42)
Generation 2.5—Mother Migrant—All Other	1.08	(1.03–1.13)	1.05	(1.00–1.10)
Generation 2.5—Father Migrant—Nordic	1.04	(1.01–1.08)	1.02	(0.98–1.05)
Generation 2.5—Father Migrant—Poland	1.03	(0.85–1.25)	1.05	(0.86–1.29)
Generation 2.5—Father Migrant—Turkey	0.98	(0.81–1.19)	1.25	(1.02–1.53)
Generation 2.5—Father Migrant—Europe South	1.06	(0.98–1.15)	1.14	(1.05–1.23)
Generation 2.5—Father Migrant—Africa North	1.25	(1.09–1.43)	1.19	(1.01–1.39)
Generation 2.5—Father Migrant—India	1.48	(1.11–1.97)	1.07	(0.77–1.49)
Generation 2.5—Father Migrant—All Other	1.08	(1.05–1.12)	1.04	(1.00–1.08)

*Note:* All HRs are controlled for period, age at previous birth, education, unemployment benefits, student allowances, and employment status.

Contrasting our findings with those for first and second births, we did not find evidence of depressed third birth rates among the majority of groups of descendants to immigrants. Among second generation Turks and North Africans, we found clear evidence of elevated third birth rates when compared with the native Swedish population. For example, third birth rates were elevated among men who are descendants of two immigrants from Turkey (HR: 1.63 (1.51–1.75)) and North Africa (HR: 1.72 (1.34–2.19)). However, no similar elevation was observed among their counterparts from exogamous relationships with a Swedish-born parent, which reveals a clear difference between the patterns of behavior in Generations 2.0 and 2.5.

A comprehensive overview of all utilized Cox models for the transition to a third birth is provided in Tables S8A and S8B. An overview of the study populations at the time of any third birth is presented in Table S9.

## Discussion

### Interpretation of Key Findings

In this study, we investigated the childbearing patterns across the generations and genders of immigrants and their descendants in Sweden. For this purpose, we drew on register data covering the demographic events for the total population in Sweden and linked these data with socioeconomic information from administrative registers. We utilized a life-course approach with time-varying covariates covering a period of over 25 years. This approach is crucial as many domains in the lives of individuals evolve dynamically over the life course. The setup allowed us to gain much better insights into behavioral patterns in relation to childbearing events than relying on aggregate descriptive statistics such as Total Fertility Rates.

Our analyses indicate elevated first birth rates shortly after arrival for most groups of immigrants who moved to Sweden as adults—this largely held for women as well as for men. In line with previous research on immigrant women in different contexts (e.g., Andersson 2004; Milewski 2007; Parrado 2011), our results for first birth also indicate that fertility rates subsequently decline rapidly with time since migration to the new context. Overall, our findings for immigrants who arrived in Sweden as adults lend support to previous observations that multiple factors related to family formation, socialization, adaptation, and selectivity are at play in shaping the fertility patterns after arrival (Kulu 2005; Milewski 2007; Andersson 2021; Lindström, Mussino and Olah 2022). For example, the patterns of fertility change in immediate relation to the migration event suggest that migration and family formation are often interrelated in the life course (Kulu and Milewski 2007), with a migration event more often occurring before becoming a parent than the other way around. A novelty of our findings is that we could demonstrate that this pattern is prevalent not only for immigrant women but also for immigrant men, reflecting that women and men often move together as couples. We also found support for the socialization hypothesis as our findings showed that fertility patterns among first-generation women and men tended to reflect patterns of their respective country-of-origin backgrounds. This was especially apparent for third births and, more generally, with respect to low-fertility backgrounds in Europe. Simultaneously, the low first-birth rates among male immigrants from India lend support to the hypothesis of migrant selectivity, reflecting that Indian migrants to Sweden are very different from the average population of that country. Changing first birth rates for adult immigrant women and men with increasing time in Sweden can be regarded as evidence of their adaptation to the new context (Andersson 2004; Mussino, Wilson and Andersson 2021).

The first-birth patterns of immigrants who arrived in Sweden as children also provide some support for the role of socialization into early and high or late and low fertility. For example, first birth rates were elevated for childhood immigrants from Turkey and North Africa and depressed for those from Poland and Southern Europe. To shed further light on the location of the 1.5 generation between the generations of immigrants and their descendants, other research has explored the role of the age at arrival in shaping the behavior of Generation 1.5—with the impact of different types of childhood socialization on behaviors throughout the life course (Wilson 2020, 2021; Mussino, Wilson and Andersson 2021). For the descendant of immigrants, we distinguished between second-generation individuals with two migrant parents and those with one foreign-born and one Swedish-born parent. For individuals with only one foreign-born parent, we differentiated whether it was the father or the mother who was an immigrant. Overall, patterns of fertility among the second generation showed some degree of similarities with patterns among the native Swedish population, indicating some extent of assimilation within the Swedish context. However, the findings also demonstrate that most groups of descendants of immigrants displayed somewhat depressed first- and second-birth rates, also in the case when only one of the parents was an immigrant. The negative associations with first birth rates were less strong for male than for female descendants of immigrants and for the descendants of a Swedish-born mother and foreign-born father. The latter finding may reflect that mothers have a somewhat larger role than fathers in the socialization of children in a given context. Similar findings for women in the second generation have previously been reported for Sweden but without distinguishing between differences in the parental composition or including men in the analyses (Andersson, Persson and Obućina 2017). The depressed first-birth rates in the second generation, in comparison to native Swedes, may point toward the detrimental role of having a minority status. According to the minority status hypothesis, depressed first birth rates could be explained by a situation in which some descendants of immigrants need to invest more resources into education and employment to achieve the same level of security as their native Swedish counterparts, leading to postponed family formation (Andersson, Persson and Obućina 2017). While we observed depressed first and second birth rates among men and women of most second-generation groups, no similar depression was found in relation to third births. In contrast, individuals of Turkish and North African origin (Generation 2.0) had elevated third-birth rates suggesting the presence of larger families among those with two immigrant parents and lending some support to the subculture hypothesis.

Despite some degrees of proximity to the native Swedish population, our results also indicate a substantial amount of heterogeneity in the fertility outcomes among immigrants and their descendants in Sweden. For example, our results showed that patterns in childbearing differed between the offspring from endogamous and exogamous relationships. In several cases, birth rates among the offspring from exogamous relationships with a Swedish-born parent tended to be closer to the native Swedish population than what held for offspring from immigrant endogamous relationships. A crucial finding is also that such a large majority of second-generation women and men stem from parental backgrounds that involve a Swedish-born person (Table 1). Mixed unions and marriages may be among the strongest mechanisms associated with immigrant integration with respect to fertility and family dynamics and future research needs to pay better attention to differences in the parental backgrounds of people who are often lumped together as belonging to a “second generation.” In addition, our results revealed that the moderating role of having a Swedish-born mother may be stronger than that of having a Swedish-born father. The exact mechanism behind these gender patterns remains unknown, but we speculate that native Swedish women have access to a wider range of social networks that could be associated with a higher degree of integration into the Swedish context and its social fabric. Nevertheless, this highlights a clear research gap, and that the role of mixed parental unions—and in particular the native component of these unions is a highly understudied concept (Braack and Milewski 2019).

Our differentiation by country-of-origin backgrounds and comparisons with research findings from other contexts reveal that first- and second-generation individuals from the same fertility context may behave very differently in different countries and welfare regimes. For example, descendants of immigrants from India typically represent a high-fertility pattern in the UK (Robards and Berrington 2016), but we found no evidence for this in the case of Sweden. To explain these differences, the hypotheses of selection may provide helpful and point toward the fact that migrants and their descendants from India have very different profiles in the two countries. In Sweden, migration from India is a relatively recent phenomenon and strongly linked to participation in education and employment (SCB 2023), while migration from India to the UK has been marked by its’ colonial and postcolonial links and produced a more heterogeneous group of migrants and their descendants (Khadria 2006). At the same time, different cultural and welfare contexts of Sweden and the UK might shape integration processes differently.

Lastly, the findings of our study highlight the gendered nature of how fertility differentials are maintained between population subgroups and generations with different migration backgrounds. There are differences between groups in the timing of becoming a mother or father and sometimes differences between migrant women and men in their fertility change shortly after migration. Further, there are differences in behavior between women and men with second-generation backgrounds and differences related to whether it was the mother or the father who was an immigrant in exogamous parental unions. For future research on fertility and family dynamics of immigrants and their descendants, it should be imperative to incorporate a strong gender perspective and to incorporate data that cover the life-course dynamics of women as well as of men.

### Limitations

Our study has many strengths but is also subject to some limitations. For example, we did not capture births which may have occurred outside of Sweden unless the children themselves had also migrated to Sweden. This means that, for immigrants who arrived in Sweden as adults, information on birth histories might in some cases be incomplete and not always reflect the true parities—for example in cases where parents moved to Sweden without their children. The omission of premigration childbearing events may be a larger cause for concern when studying men than when studying women, but we cannot quantify the extent of any such bias.

Further, our synthetic cohort design and its inherent period perspective when studying events during 1991–2017 implies that the birth cohorts 1941–2000 are not equally represented among the at-risk populations. For example, some individuals of the older birth cohorts will have experienced their first birth before the start of the study period and do not form part of our study population.

We covered several dominant country-of-origin backgrounds in our study. However, due to the sheer number of countries of origin in Sweden, we were not able to capture all possible cases. We instead combined all remaining groups into a residual “other” category. As shown in Supplemental Tables S10A, S10B, and S10C, this “other” category is not dominated by any particular nationality, further evidencing a considerable amount of heterogeneity in the immigrant population in Sweden. Heterogeneity is also manifested in other country groupings that we cover in our study and it is a limitation is that we did not fully account for this heterogeneity. Here, the use of one overarching “Nordic” population subgroup may be a particularly pressing case. Despite long-standing similarities in welfare state regimes and cultural norms, the patterns of migration from Finland differ somewhat from those of Denmark, Norway, and Iceland. A disaggregated analysis of the fertility outcomes of immigrants and their descendants from individual Nordic countries reveals relatively depressed first-birth rates of immigrant men and women from Finland as compared to those from the other Nordic countries. To the benefit of the reader, we display those disaggregated results in our Supplemental Tables S11a and b. In contrast, patterns were very similar for the descendants of immigrants from the different Nordic countries (Tables S11c, d and e).

While our models for first births reflect unconditional HRs, our models for second and third births reflect differences in conditional HRs, given that an individual has already become the father or mother of a previous child. This means that all presented HRs of the models for second and third births are conditional upon individuals having experienced a birth of the previous parity. This type of event-history approach implies that we cannot firmly distinguish between the quantum and timing of the tendency to have another child. While Cox proportional hazards models allow for statements about differences in event intensities, no statements can be made about the exact absolute magnitude of the underlying birth rates. However, the concepts of relative rates, relative risks, and HRs still allow for a sound comparison of differences in underlying event rates—given they are interpreted correctly in relation to a suitable reference category and are not mistakenly interpreted as birth rates themselves.

## Conclusion

In our study, we examined childbearing across the generations and genders of immigrants and their descendants in comparison with the native-born population in Sweden. We found evidence that fertility among second-generation women and men is drifting away from patterns observed among their first-generation counterparts and that patterns sometimes differ between the offspring from endogamous and exogamous parental unions. In general, the second generation exhibited depressed levels of first and second births when compared to the native Swedish population. Our findings highlight that second-generation individuals do not represent a homogeneous group. Future research with a particular focus on the descendants of immigrants should aim to account for this diversity, including the gendered nature of different patterns of family-related outcomes.

## Supplemental Material

sj-docx-1-mrx-10.1177_01979183241245072 - Supplemental material for Childbearing Across Immigrants and Their Descendants in Sweden: The Role of Generation and Gender Supplemental material, sj-docx-1-mrx-10.1177_01979183241245072 for Childbearing Across Immigrants and Their Descendants in Sweden: The Role of Generation and Gender by Andreas Höhn, Hill Kulu, Gunnar Andersson and Brad Campbell in International Migration Review
