# Supplementary material for: Childbearing Across Immigrants and Their Descendants in Sweden: The Role of Generation and Gender
Source: Int Migr Rev. 2024 Apr 25;59(4):1919–49. doi: 10.1177/01979183241245072 (PMC13020918; doi:10.1177/01979183241245072)
Supplement: sj-docx-1-mrx-10.1177_01979183241245072 - Supplemental material for Childbearing Across Immigrants and Their Descendants in Sweden: The Role of Generation and Gender [file sj-docx-1-mrx-10.1177_01979183241245072.docx]

# Supplementary Material: Childbearing Across the Generations of Immigrants and their Descendants in Sweden: A Register-Based Study

## First Birth

**S-1 Table:** Overview of the study population at risk of a first birth by sex, population subgroup, and country-of-origin background.

| **Population Subgroup** | **N Males** | **N Females** | **N Total** | **N Total (%)** | **Males Birth** | **Females Birth** |
| --- | --- | --- | --- | --- | --- | --- |
| Native Swedish | 1858844 | 1605907 | 3464751 | 65.10 | 815448 | 827415 |
| Generation 1.0 - Nordic | 65306 | 57343 | 122649 | 2.30 | 10749 | 12946 |
| Generation 1.0 - Poland | 26080 | 21484 | 47564 | 0.89 | 5327 | 8054 |
| Generation 1.0 - Turkey | 14615 | 8896 | 23511 | 0.44 | 7087 | 5756 |
| Generation 1.0 - Europe South | 19204 | 12077 | 31281 | 0.59 | 3777 | 2733 |
| Generation 1.0 - Africa North | 12087 | 7211 | 19298 | 0.36 | 4916 | 4668 |
| Generation 1.0 - India | 16015 | 6862 | 22877 | 0.43 | 2263 | 2201 |
| Generation 1.0 - All Other | 381540 | 294040 | 675580 | 12.69 | 118728 | 125120 |
| Generation 1.5 - Nordic | 20386 | 16091 | 36477 | 0.69 | 8116 | 7341 |
| Generation 1.5 - Poland | 6187 | 5699 | 11886 | 0.22 | 2138 | 2338 |
| Generation 1.5 - Turkey | 4745 | 4127 | 8872 | 0.17 | 2674 | 2457 |
| Generation 1.5 - Europe South | 2377 | 1870 | 4247 | 0.08 | 866 | 663 |
| Generation 1.5 - Africa North | 972 | 787 | 1759 | 0.03 | 278 | 304 |
| Generation 1.5 - India | 2539 | 4876 | 7415 | 0.14 | 939 | 2521 |
| Generation 1.5 - All Other | 122395 | 111847 | 234242 | 4.40 | 38111 | 45063 |
| Generation 2.0 - Nordic | 31045 | 26571 | 57616 | 1.08 | 15906 | 16520 |
| Generation 2.0 - Poland | 3011 | 2865 | 5876 | 0.11 | 863 | 1003 |
| Generation 2.0 - Turkey | 9024 | 8580 | 17604 | 0.33 | 2896 | 3554 |
| Generation 2.0 - Europe South | 2750 | 2557 | 5307 | 0.10 | 1273 | 1190 |
| Generation 2.0 - Africa North | 1866 | 1808 | 3674 | 0.07 | 345 | 498 |
| Generation 2.0 - India | 652 | 652 | 1304 | 0.02 | 87 | 110 |
| Generation 2.0 - All Other | 70810 | 65606 | 136416 | 2.56 | 16031 | 18143 |
| Generation 2.5 - Mother Migrant - Nordic | 59281 | 50203 | 109484 | 2.06 | 25904 | 26245 |
| Generation 2.5 - Mother Migrant - Poland | 4661 | 4360 | 9021 | 0.17 | 1472 | 1731 |
| Generation 2.5 - Mother Migrant - Turkey | 301 | 271 | 572 | 0.01 | 50 | 57 |
| Generation 2.5 - Mother Migrant - Europe South | 1834 | 1708 | 3542 | 0.07 | 710 | 777 |
| Generation 2.5 - Mother Migrant - Africa North | 236 | 250 | 486 | 0.01 | 54 | 74 |
| Generation 2.5 - Mother Migrant - India | 528 | 521 | 1049 | 0.02 | 99 | 109 |
| Generation 2.5 - Mother Migrant - All Other | 32088 | 28325 | 60413 | 1.14 | 11081 | 11077 |
| Generation 2.5 - Father Migrant - Nordic | 43274 | 38638 | 81912 | 1.54 | 18956 | 20326 |
| Generation 2.5 - Father Migrant - Poland | 1641 | 1566 | 3207 | 0.06 | 606 | 648 |
| Generation 2.5 - Father Migrant - Turkey | 1858 | 1687 | 3545 | 0.07 | 500 | 606 |
| Generation 2.5 - Father Migrant - Europe South | 7235 | 6775 | 14010 | 0.26 | 3159 | 3467 |
| Generation 2.5 - Father Migrant - Africa North | 2757 | 2630 | 5387 | 0.10 | 895 | 1081 |
| Generation 2.5 - Father Migrant - India | 587 | 513 | 1100 | 0.02 | 204 | 198 |
| Generation 2.5 - Father Migrant - All Other | 45864 | 42444 | 88308 | 1.66 | 17387 | 18798 |

**S-2A Table:** Comprehensive overview of results of multivariate cox proportional hazards models for transitions to first birth for females, differentiated by country-of-origin background.

| **Parameter** | **Hazard Ratio** | **CI 95lower** | **CI 95upper** |
| --- | --- | --- | --- |
| Generation 1.0 - Nordic (0,2] (Ref: Native Swedish) | 1.08 | 1.05 | 1.11 |
| Generation 1.0 - Nordic (2,5] | 1.17 | 1.14 | 1.21 |
| Generation 1.0 - Nordic (5,Inf] | 0.86 | 0.83 | 0.88 |
| Generation 1.0 - Poland (0,2] | 1.84 | 1.78 | 1.90 |
| Generation 1.0 - Poland (2,5] | 1.36 | 1.30 | 1.41 |
| Generation 1.0 - Poland (5,Inf] | 0.95 | 0.91 | 0.99 |
| Generation 1.0 - Turkey (0,2] | 5.62 | 5.43 | 5.81 |
| Generation 1.0 - Turkey (2,5] | 2.88 | 2.73 | 3.03 |
| Generation 1.0 - Turkey (5,Inf] | 1.37 | 1.29 | 1.46 |
| Generation 1.0 - Europe South (0,2] | 0.63 | 0.59 | 0.68 |
| Generation 1.0 - Europe South (2,5] | 0.84 | 0.79 | 0.89 |
| Generation 1.0 - Europe South (5,Inf] | 0.89 | 0.83 | 0.95 |
| Generation 1.0 - Africa North (0,2] | 5.93 | 5.73 | 6.13 |
| Generation 1.0 - Africa North (2,5] | 2.52 | 2.36 | 2.70 |
| Generation 1.0 - Africa North (5,Inf] | 1.44 | 1.31 | 1.57 |
| Generation 1.0 - India (0,2] | 1.97 | 1.86 | 2.09 |
| Generation 1.0 - India (2,5] | 1.69 | 1.57 | 1.82 |
| Generation 1.0 - India (5,Inf] | 1.12 | 1.00 | 1.26 |
| Generation 1.0 - All Other (0,2] | 2.48 | 2.45 | 2.50 |
| Generation 1.0 - All Other (2,5] | 1.56 | 1.54 | 1.58 |
| Generation 1.0 - All Other (5,Inf] | 1.09 | 1.08 | 1.11 |
| Generation 1.5 - Nordic | 0.97 | 0.95 | 0.99 |
| Generation 1.5 - Poland | 0.89 | 0.85 | 0.92 |
| Generation 1.5 - Turkey | 1.29 | 1.24 | 1.35 |
| Generation 1.5 - Europe South | 0.76 | 0.70 | 0.82 |
| Generation 1.5 - Africa North | 1.21 | 1.08 | 1.36 |
| Generation 1.5 - India | 0.79 | 0.76 | 0.82 |
| Generation 1.5 - All Other | 1.03 | 1.02 | 1.04 |
| Generation 2.0 - Nordic | 1.04 | 1.02 | 1.05 |
| Generation 2.0 - Poland | 0.72 | 0.68 | 0.77 |
| Generation 2.0 - Turkey | 1.07 | 1.03 | 1.10 |
| Generation 2.0 - Europe South | 0.73 | 0.69 | 0.78 |
| Generation 2.0 - Africa North | 0.84 | 0.77 | 0.92 |
| Generation 2.0 - India | 0.42 | 0.35 | 0.50 |
| Generation 2.0 - All Other | 0.81 | 0.80 | 0.83 |
| Generation 2.5 - Mother Migrant - Nordic | 0.96 | 0.95 | 0.97 |
| Generation 2.5 - Mother Migrant - Poland | 0.76 | 0.73 | 0.80 |
| Generation 2.5 - Mother Migrant - Turkey | 0.78 | 0.60 | 1.01 |
| Generation 2.5 - Mother Migrant - Europe South | 0.84 | 0.79 | 0.91 |
| Generation 2.5 - Mother Migrant - Africa North | 0.68 | 0.54 | 0.86 |
| Generation 2.5 - Mother Migrant - India | 0.72 | 0.60 | 0.87 |
| Generation 2.5 - Mother Migrant - All Other | 0.83 | 0.82 | 0.85 |
| Generation 2.5 - Father Migrant - Nordic | 1.04 | 1.03 | 1.05 |
| Generation 2.5 - Father Migrant - Poland | 0.87 | 0.81 | 0.94 |
| Generation 2.5 - Father Migrant - Turkey | 0.88 | 0.82 | 0.96 |
| Generation 2.5 - Father Migrant - Europe South | 0.88 | 0.85 | 0.91 |
| Generation 2.5 - Father Migrant - Africa North | 0.86 | 0.81 | 0.91 |
| Generation 2.5 - Father Migrant - India | 0.76 | 0.66 | 0.88 |
| Generation 2.5 - Father Migrant - All Other | 0.89 | 0.87 | 0.90 |
| 1995-1998 (Ref: 1991-1994) | 0.83 | 0.83 | 0.84 |
| 1999-2002 | 0.82 | 0.81 | 0.83 |
| 2003-2006 | 0.88 | 0.87 | 0.88 |
| 2007-2010 | 0.90 | 0.89 | 0.90 |
| 2011-2014 | 0.83 | 0.83 | 0.84 |
| 2015-2017 | 0.77 | 0.77 | 0.78 |
| Education Secondary (Ref: Primary) | 0.85 | 0.84 | 0.85 |
| Education Tertiary | 0.92 | 0.91 | 0.92 |
| Education Missing | 0.67 | 0.66 | 0.68 |
| Unemployment Benefits: Yes (Ref: No) | 1.12 | 1.12 | 1.13 |
| Student Benefits: Yes (Ref: No) | 0.39 | 0.39 | 0.39 |
| In Employment: No (Ref: Yes) | 0.68 | 0.68 | 0.69 |

**S-2B Table:** Comprehensive overview of results of multivariate cox proportional hazards models for transitions to first birth for males, differentiated by country-of-origin background.

| **Parameter** | **Hazard Ratio** | **CI 95lower** | **CI 95upper** |
| --- | --- | --- | --- |
| Generation 1.0 - Nordic (0,2] (Ref: Native Swedish) | 1.63 | 1.57 | 1.68 |
| Generation 1.0 - Nordic (2,5] | 1.40 | 1.35 | 1.45 |
| Generation 1.0 - Nordic (5,Inf] | 0.84 | 0.82 | 0.87 |
| Generation 1.0 - Poland (0,2] | 1.18 | 1.12 | 1.23 |
| Generation 1.0 - Poland (2,5] | 1.22 | 1.16 | 1.27 |
| Generation 1.0 - Poland (5,Inf] | 1.02 | 0.97 | 1.07 |
| Generation 1.0 - Turkey (0,2] | 3.93 | 3.79 | 4.08 |
| Generation 1.0 - Turkey (2,5] | 1.88 | 1.80 | 1.97 |
| Generation 1.0 - Turkey (5,Inf] | 1.78 | 1.71 | 1.86 |
| Generation 1.0 - Europe South (0,2] | 0.96 | 0.90 | 1.02 |
| Generation 1.0 - Europe South (2,5] | 0.96 | 0.90 | 1.01 |
| Generation 1.0 - Europe South (5,Inf] | 0.99 | 0.94 | 1.04 |
| Generation 1.0 - Africa North (0,2] | 2.37 | 2.26 | 2.48 |
| Generation 1.0 - Africa North (2,5] | 1.11 | 1.05 | 1.17 |
| Generation 1.0 - Africa North (5,Inf] | 1.77 | 1.69 | 1.85 |
| Generation 1.0 - India (0,2] | 1.00 | 0.93 | 1.07 |
| Generation 1.0 - India (2,5] | 0.81 | 0.75 | 0.88 |
| Generation 1.0 - India (5,Inf] | 1.18 | 1.09 | 1.26 |
| Generation 1.0 - All Other (0,2] | 1.78 | 1.76 | 1.80 |
| Generation 1.0 - All Other (2,5] | 1.52 | 1.50 | 1.54 |
| Generation 1.0 - All Other (5,Inf] | 1.47 | 1.46 | 1.49 |
| Generation 1.5 - Nordic | 0.91 | 0.89 | 0.93 |
| Generation 1.5 - Poland | 0.95 | 0.91 | 1.00 |
| Generation 1.5 - Turkey | 1.58 | 1.52 | 1.64 |
| Generation 1.5 - Europe South | 0.98 | 0.92 | 1.05 |
| Generation 1.5 - Africa North | 1.12 | 1.00 | 1.26 |
| Generation 1.5 - India | 0.68 | 0.64 | 0.72 |
| Generation 1.5 - All Other | 1.07 | 1.06 | 1.08 |
| Generation 2.0 - Nordic | 0.97 | 0.95 | 0.98 |
| Generation 2.0 - Poland | 0.84 | 0.79 | 0.90 |
| Generation 2.0 - Turkey | 1.11 | 1.07 | 1.15 |
| Generation 2.0 - Europe South | 0.89 | 0.84 | 0.94 |
| Generation 2.0 - Africa North | 0.88 | 0.80 | 0.98 |
| Generation 2.0 - India | 0.49 | 0.40 | 0.61 |
| Generation 2.0 - All Other | 0.89 | 0.88 | 0.91 |
| Generation 2.5 - Mother Migrant - Nordic | 0.93 | 0.92 | 0.95 |
| Generation 2.5 - Mother Migrant - Poland | 0.81 | 0.77 | 0.85 |
| Generation 2.5 - Mother Migrant - Turkey | 0.84 | 0.64 | 1.11 |
| Generation 2.5 - Mother Migrant - Europe South | 1.00 | 0.93 | 1.07 |
| Generation 2.5 - Mother Migrant - Africa North | 0.76 | 0.58 | 0.99 |
| Generation 2.5 - Mother Migrant - India | 0.86 | 0.70 | 1.04 |
| Generation 2.5 - Mother Migrant - All Other | 0.91 | 0.89 | 0.93 |
| Generation 2.5 - Father Migrant - Nordic | 1.01 | 0.99 | 1.02 |
| Generation 2.5 - Father Migrant - Poland | 0.96 | 0.88 | 1.04 |
| Generation 2.5 - Father Migrant - Turkey | 0.97 | 0.89 | 1.06 |
| Generation 2.5 - Father Migrant - Europe South | 0.95 | 0.92 | 0.98 |
| Generation 2.5 - Father Migrant - Africa North | 0.90 | 0.84 | 0.96 |
| Generation 2.5 - Father Migrant - India | 0.84 | 0.74 | 0.97 |
| Generation 2.5 - Father Migrant - All Other | 0.95 | 0.93 | 0.96 |
| 1995-1998 (Ref: 1991-1994) | 0.82 | 0.81 | 0.82 |
| 1999-2002 | 0.78 | 0.78 | 0.79 |
| 2003-2006 | 0.86 | 0.85 | 0.87 |
| 2007-2010 | 0.90 | 0.89 | 0.91 |
| 2011-2014 | 0.84 | 0.84 | 0.85 |
| 2015-2017 | 0.77 | 0.76 | 0.77 |
| Education Secondary (Ref: Primary) | 1.00 | 0.99 | 1.00 |
| Education Tertiary | 1.03 | 1.03 | 1.04 |
| Education Missing | 0.59 | 0.58 | 0.60 |
| Unemployment Benefits: Yes (Ref: No) | 1.08 | 1.07 | 1.09 |
| Student Benefits: Yes (Ref: No) | 0.57 | 0.56 | 0.57 |
| In Employment: No (Ref: Yes) | 0.51 | 0.50 | 0.51 |

**S-3 Table:** Overview of sociodemographic characteristics for the study population at time of first birth.

| **Characteristic** | **N** | **in %** |
| --- | --- | --- |
| **Sex** |  |  |
| Males | 1,139,895 | 49.22 |
| Females | 1,175,792 | 50.78 |
| **Population Subgroup** |  |  |
| Native Swedish | 1,642,863 | 70.94 |
| Generation 1.0 | 314,325 | 13.57 |
| Generation 1.5 | 113,809 | 4.91 |
| Generation 2.0 | 78,419 | 3.39 |
| Generation 2.5 - Mother Migrant | 79,440 | 3.43 |
| Generation 2.5 - Father Migrant | 86,831 | 3.75 |
| **Birth Cohorts** |  |  |
| 1940-1949 | 3,063 | 0.13 |
| 1950-1959 | 82,541 | 3.56 |
| 1960-1969 | 594,521 | 25.67 |
| 1970-1979 | 888,321 | 38.36 |
| 1980-1989 | 635,810 | 27.46 |
| 1990-1999 | 111,431 | 4.81 |
| Age in years: Median (IQR) | 29.21 | (25.79 - 32.75) |
| **Education** |  |  |
| Education Primary | 271,805 | 11.74 |
| Education Secondary | 1,089,090 | 47.03 |
| Education Tertiary | 905,989 | 39.12 |
| Education Missing | 48,803 | 2.11 |
| **Benefits** |  |  |
| Student No | 2,075,490 | 89.63 |
| Student Yes | 240,197 | 10.37 |
| Unemployed No | 2,017,130 | 87.11 |
| Unemployed Yes | 298,557 | 12.89 |
| **Employment** |  |  |
| Employed Yes | 1,867,594 | 80.65 |
| Employed No | 448,093 | 19.35 |

## Second Birth

**S-4 Table:** Overview of the study population at risk of a second birth by sex, population subgroup, and country-of-origin background.

| **Population Subgroup** | **N Males** | **N Females** | **N Total** | **N Total (%)** | **Males Birth** | **Females Birth** |
| --- | --- | --- | --- | --- | --- | --- |
| Native Swedish | 802715 | 814752 | 1617467 | 70.92 | 571456 | 597714 |
| Generation 1.0 - Nordic | 10585 | 12720 | 23305 | 1.02 | 5537 | 7007 |
| Generation 1.0 - Poland | 5259 | 7956 | 13215 | 0.58 | 2296 | 3736 |
| Generation 1.0 - Turkey | 6959 | 5659 | 12618 | 0.55 | 4749 | 4043 |
| Generation 1.0 - Europe South | 3714 | 2698 | 6412 | 0.28 | 1956 | 1306 |
| Generation 1.0 - Africa North | 4853 | 4591 | 9444 | 0.41 | 3156 | 3107 |
| Generation 1.0 - India | 2227 | 2170 | 4397 | 0.19 | 822 | 868 |
| Generation 1.0 - All Other | 117143 | 123365 | 240508 | 10.55 | 73703 | 73085 |
| Generation 1.5 - Nordic | 7981 | 7221 | 15202 | 0.67 | 5561 | 5063 |
| Generation 1.5 - Poland | 2108 | 2299 | 4407 | 0.19 | 1300 | 1440 |
| Generation 1.5 - Turkey | 2634 | 2413 | 5047 | 0.22 | 2144 | 1897 |
| Generation 1.5 - Europe South | 856 | 649 | 1505 | 0.07 | 618 | 429 |
| Generation 1.5 - Africa North | 273 | 297 | 570 | 0.02 | 182 | 202 |
| Generation 1.5 - India | 931 | 2491 | 3422 | 0.15 | 626 | 1650 |
| Generation 1.5 - All Other | 37617 | 44495 | 82112 | 3.60 | 24129 | 29250 |
| Generation 2.0 - Nordic | 15706 | 16259 | 31965 | 1.40 | 11022 | 11968 |
| Generation 2.0 - Poland | 852 | 988 | 1840 | 0.08 | 521 | 587 |
| Generation 2.0 - Turkey | 2857 | 3509 | 6366 | 0.28 | 1997 | 2453 |
| Generation 2.0 - Europe South | 1259 | 1180 | 2439 | 0.11 | 883 | 780 |
| Generation 2.0 - Africa North | 338 | 493 | 831 | 0.04 | 204 | 279 |
| Generation 2.0 - India | 85 | 110 | 195 | 0.01 | 50 | 69 |
| Generation 2.0 - All Other | 15801 | 17856 | 33657 | 1.48 | 10246 | 11310 |
| Generation 2.5 - Mother Migrant - Nordic | 25505 | 25855 | 51360 | 2.25 | 17656 | 18554 |
| Generation 2.5 - Mother Migrant - Poland | 1443 | 1708 | 3151 | 0.14 | 908 | 1111 |
| Generation 2.5 - Mother Migrant - Turkey | 49 | 55 | 104 | < 0.01 | 27 | 28 |
| Generation 2.5 - Mother Migrant - Europe South | 702 | 762 | 1464 | 0.06 | 469 | 527 |
| Generation 2.5 - Mother Migrant - Africa North | 51 | 72 | 123 | 0.01 | 30 | 39 |
| Generation 2.5 - Mother Migrant - India | 98 | 107 | 205 | 0.01 | 63 | 64 |
| Generation 2.5 - Mother Migrant - All Other | 10894 | 10889 | 21783 | 0.96 | 7498 | 7448 |
| Generation 2.5 - Father Migrant - Nordic | 18689 | 20029 | 38718 | 1.70 | 12791 | 14179 |
| Generation 2.5 - Father Migrant - Poland | 594 | 634 | 1228 | 0.05 | 381 | 412 |
| Generation 2.5 - Father Migrant - Turkey | 492 | 598 | 1090 | 0.05 | 294 | 377 |
| Generation 2.5 - Father Migrant - Europe South | 3111 | 3404 | 6515 | 0.29 | 2083 | 2344 |
| Generation 2.5 - Father Migrant - Africa North | 887 | 1061 | 1948 | 0.09 | 552 | 692 |
| Generation 2.5 - Father Migrant - India | 202 | 197 | 399 | 0.02 | 145 | 138 |
| Generation 2.5 - Father Migrant - All Other | 17093 | 18479 | 35572 | 1.56 | 11906 | 12721 |

**S-5A Table:** Results of multivariate cox proportional hazards models for transitions to second birth for females, differentiated by country-of-origin background.

| **Parameter** | **Hazard Ratio** | **CI 95lower** | **CI 95upper** |
| --- | --- | --- | --- |
| Generation 1.0 - Nordic (Ref: Native Swedish) | 0.93 | 0.90 | 0.95 |
| Generation 1.0 - Poland | 0.53 | 0.52 | 0.55 |
| Generation 1.0 - Turkey | 0.99 | 0.96 | 1.02 |
| Generation 1.0 - Europe South | 0.86 | 0.82 | 0.91 |
| Generation 1.0 - Africa North | 1.27 | 1.22 | 1.31 |
| Generation 1.0 - India | 0.62 | 0.58 | 0.66 |
| Generation 1.0 - All Other | 0.92 | 0.91 | 0.92 |
| Generation 1.5 - Nordic | 0.84 | 0.82 | 0.87 |
| Generation 1.5 - Poland | 0.76 | 0.72 | 0.80 |
| Generation 1.5 - Turkey | 0.99 | 0.95 | 1.04 |
| Generation 1.5 - Europe South | 0.90 | 0.82 | 0.99 |
| Generation 1.5 - Africa North | 0.93 | 0.81 | 1.07 |
| Generation 1.5 - India | 0.78 | 0.75 | 0.82 |
| Generation 1.5 - All Other | 0.88 | 0.87 | 0.89 |
| Generation 2.0 - Nordic | 0.91 | 0.89 | 0.92 |
| Generation 2.0 - Poland | 0.83 | 0.77 | 0.90 |
| Generation 2.0 - Turkey | 0.96 | 0.92 | 1.00 |
| Generation 2.0 - Europe South | 0.92 | 0.86 | 0.99 |
| Generation 2.0 - Africa North | 0.97 | 0.87 | 1.10 |
| Generation 2.0 - India | 0.99 | 0.78 | 1.26 |
| Generation 2.0 - All Other | 0.93 | 0.92 | 0.95 |
| Generation 2.5 - Mother Migrant - Nordic | 0.96 | 0.94 | 0.97 |
| Generation 2.5 - Mother Migrant - Poland | 0.90 | 0.85 | 0.95 |
| Generation 2.5 - Mother Migrant - Turkey | 0.76 | 0.52 | 1.10 |
| Generation 2.5 - Mother Migrant - Europe South | 0.92 | 0.84 | 1.00 |
| Generation 2.5 - Mother Migrant - Africa North | 0.84 | 0.61 | 1.15 |
| Generation 2.5 - Mother Migrant - India | 1.12 | 0.87 | 1.42 |
| Generation 2.5 - Mother Migrant - All Other | 0.97 | 0.94 | 0.99 |
| Generation 2.5 - Father Migrant - Nordic | 0.93 | 0.91 | 0.94 |
| Generation 2.5 - Father Migrant - Poland | 0.93 | 0.84 | 1.02 |
| Generation 2.5 - Father Migrant - Turkey | 0.89 | 0.80 | 0.98 |
| Generation 2.5 - Father Migrant - Europe South | 0.92 | 0.88 | 0.96 |
| Generation 2.5 - Father Migrant - Africa North | 0.93 | 0.86 | 1.00 |
| Generation 2.5 - Father Migrant - India | 1.01 | 0.85 | 1.19 |
| Generation 2.5 - Father Migrant - All Other | 0.94 | 0.92 | 0.95 |
| 1995-1998 (Ref: 1991-1994) | 0.86 | 0.85 | 0.87 |
| 1999-2002 | 0.89 | 0.88 | 0.90 |
| 2003-2006 | 0.96 | 0.95 | 0.97 |
| 2007-2010 | 0.95 | 0.94 | 0.96 |
| 2011-2014 | 0.91 | 0.90 | 0.92 |
| 2015-2017 | 0.90 | 0.89 | 0.91 |
| Age at Previous Birth | 0.94 | 0.94 | 0.94 |
| Education Secondary (Ref: Primary) | 1.31 | 1.30 | 1.32 |
| Education Tertiary | 1.85 | 1.83 | 1.86 |
| Education Missing | 1.43 | 1.40 | 1.46 |
| Unemployment Benefits: Yes (Ref: No) | 0.91 | 0.90 | 0.91 |
| Student Benefits: Yes (Ref: No) | 0.53 | 0.52 | 0.53 |
| In Employment: No (Ref: Yes) | 0.90 | 0.89 | 0.90 |

**S-5B Table:** Results of multivariate cox proportional hazards models for transitions to second birth for males, differentiated by country-of-origin background.

| **Parameter** | **Hazard Ratio** | **CI 95lower** | **CI 95upper** |
| --- | --- | --- | --- |
| Generation 1.0 - Nordic (Ref: Native Swedish) | 0.95 | 0.93 | 0.98 |
| Generation 1.0 - Poland | 0.59 | 0.57 | 0.62 |
| Generation 1.0 - Turkey | 1.00 | 0.97 | 1.03 |
| Generation 1.0 - Europe South | 0.96 | 0.92 | 1.00 |
| Generation 1.0 - Africa North | 1.13 | 1.09 | 1.17 |
| Generation 1.0 - India | 0.61 | 0.57 | 0.66 |
| Generation 1.0 - All Other | 1.09 | 1.08 | 1.09 |
| Generation 1.5 - Nordic | 0.89 | 0.87 | 0.91 |
| Generation 1.5 - Poland | 0.77 | 0.73 | 0.81 |
| Generation 1.5 - Turkey | 1.19 | 1.14 | 1.25 |
| Generation 1.5 - Europe South | 1.03 | 0.95 | 1.12 |
| Generation 1.5 - Africa North | 1.10 | 0.95 | 1.27 |
| Generation 1.5 - India | 0.90 | 0.84 | 0.98 |
| Generation 1.5 - All Other | 0.99 | 0.98 | 1.00 |
| Generation 2.0 - Nordic | 0.90 | 0.88 | 0.92 |
| Generation 2.0 - Poland | 0.92 | 0.84 | 1.00 |
| Generation 2.0 - Turkey | 1.21 | 1.16 | 1.27 |
| Generation 2.0 - Europe South | 1.05 | 0.98 | 1.12 |
| Generation 2.0 - Africa North | 1.11 | 0.97 | 1.27 |
| Generation 2.0 - India | 1.16 | 0.88 | 1.53 |
| Generation 2.0 - All Other | 0.98 | 0.96 | 1.00 |
| Generation 2.5 - Mother Migrant - Nordic | 0.94 | 0.92 | 0.95 |
| Generation 2.5 - Mother Migrant - Poland | 0.91 | 0.85 | 0.97 |
| Generation 2.5 - Mother Migrant - Turkey | 0.78 | 0.53 | 1.13 |
| Generation 2.5 - Mother Migrant - Europe South | 0.88 | 0.80 | 0.96 |
| Generation 2.5 - Mother Migrant - Africa North | 1.33 | 0.93 | 1.91 |
| Generation 2.5 - Mother Migrant - India | 0.90 | 0.71 | 1.16 |
| Generation 2.5 - Mother Migrant - All Other | 0.96 | 0.94 | 0.98 |
| Generation 2.5 - Father Migrant - Nordic | 0.92 | 0.91 | 0.94 |
| Generation 2.5 - Father Migrant - Poland | 0.91 | 0.82 | 1.00 |
| Generation 2.5 - Father Migrant - Turkey | 0.83 | 0.74 | 0.93 |
| Generation 2.5 - Father Migrant - Europe South | 0.90 | 0.86 | 0.94 |
| Generation 2.5 - Father Migrant - Africa North | 0.94 | 0.87 | 1.02 |
| Generation 2.5 - Father Migrant - India | 1.07 | 0.91 | 1.26 |
| Generation 2.5 - Father Migrant - All Other | 0.96 | 0.95 | 0.98 |
| 1995-1998 (Ref: 1991-1994) | 0.81 | 0.80 | 0.82 |
| 1999-2002 | 0.80 | 0.79 | 0.80 |
| 2003-2006 | 0.87 | 0.86 | 0.88 |
| 2007-2010 | 0.87 | 0.86 | 0.88 |
| 2011-2014 | 0.86 | 0.85 | 0.87 |
| 2015-2017 | 0.84 | 0.83 | 0.85 |
| Age at Previous Birth | 0.97 | 0.97 | 0.97 |
| Education Secondary (Ref: Primary) | 1.14 | 1.13 | 1.15 |
| Education Tertiary | 1.54 | 1.53 | 1.55 |
| Education Missing | 1.07 | 1.05 | 1.10 |
| Unemployment Benefits: Yes (Ref: No) | 0.90 | 0.89 | 0.91 |
| Student Benefits: Yes (Ref: No) | 0.84 | 0.83 | 0.85 |
| In Employment: No (Ref: Yes) | 0.75 | 0.74 | 0.75 |

**S-6 Table:** Overview of sociodemographic characteristics for the study population at time of second birth.

| **Characteristic** | **N** | **in %** |
| --- | --- | --- |
| **Sex** |  |  |
| Males | 777,961 | 48.78 |
| Females | 816,877 | 51.22 |
| **Population Subgroup** |  |  |
| Native Swedish | 1,169,170 | 73.31 |
| Generation 1.0 | 185,371 | 11.62 |
| Generation 1.5 | 74,491 | 4.67 |
| Generation 2.0 | 52,369 | 3.28 |
| Generation 2.5 - Mother Migrant | 54,422 | 3.41 |
| Generation 2.5 - Father Migrant | 59,015 | 3.7 |
| **Birth Cohorts** |  |  |
| 1940-1949 | 835 | 0.05 |
| 1950-1959 | 44,323 | 2.78 |
| 1960-1969 | 444,306 | 27.86 |
| 1970-1979 | 689,758 | 43.25 |
| 1980-1989 | 381,562 | 23.92 |
| 1990-1999 | 34,054 | 2.14 |
| Age in years: Median (IQR) | 31.80 | (28.53 - 35.08) |
| **Education** |  |  |
| Education Primary | 162,776 | 10.21 |
| Education Secondary | 753,294 | 47.23 |
| Education Tertiary | 662,845 | 41.56 |
| Education Missing | 15,923 | 1 |
| **Benefits** |  |  |
| Student No | 1,500,908 | 94.11 |
| Student Yes | 93,930 | 5.89 |
| Unemployed No | 1,396,820 | 87.58 |
| Unemployed Yes | 198,018 | 12.42 |
| **Employment** |  |  |
| Employed Yes | 1,338,806 | 83.95 |
| Employed No | 256,032 | 16.05 |

## Third Birth

**S-7 Table:** Overview of the study population at risk of a third birth by sex, population subgroup, and country-of-origin background

| **Population Subgroup** | **N Males** | **N Females** | **N Total** | **N Total (%)** | **Males Birth** | **Females Birth** |
| --- | --- | --- | --- | --- | --- | --- |
| Native Swedish | 563445 | 589435 | 1152880 | 73.29 | 150050 | 156699 |
| Generation 1.0 - Nordic | 5462 | 6886 | 12348 | 0.78 | 1245 | 1608 |
| Generation 1.0 - Poland | 2266 | 3697 | 5963 | 0.38 | 343 | 575 |
| Generation 1.0 - Turkey | 4683 | 3988 | 8671 | 0.55 | 1950 | 1657 |
| Generation 1.0 - Europe South | 1925 | 1285 | 3210 | 0.20 | 382 | 215 |
| Generation 1.0 - Africa North | 3118 | 3068 | 6186 | 0.39 | 1499 | 1350 |
| Generation 1.0 - India | 812 | 863 | 1675 | 0.11 | 161 | 153 |
| Generation 1.0 - All Other | 72804 | 72228 | 145032 | 9.22 | 27131 | 24458 |
| Generation 1.5 - Nordic | 5467 | 4994 | 10461 | 0.67 | 1679 | 1494 |
| Generation 1.5 - Poland | 1287 | 1420 | 2707 | 0.17 | 343 | 358 |
| Generation 1.5 - Turkey | 2114 | 1870 | 3984 | 0.25 | 1160 | 946 |
| Generation 1.5 - Europe South | 609 | 422 | 1031 | 0.07 | 179 | 113 |
| Generation 1.5 - Africa North | 181 | 197 | 378 | 0.02 | 76 | 81 |
| Generation 1.5 - India | 615 | 1630 | 2245 | 0.14 | 160 | 408 |
| Generation 1.5 - All Other | 23867 | 28909 | 52776 | 3.36 | 8025 | 9525 |
| Generation 2.0 - Nordic | 10878 | 11795 | 22673 | 1.44 | 3332 | 3661 |
| Generation 2.0 - Poland | 517 | 579 | 1096 | 0.07 | 123 | 129 |
| Generation 2.0 - Turkey | 1973 | 2423 | 4396 | 0.28 | 745 | 999 |
| Generation 2.0 - Europe South | 870 | 769 | 1639 | 0.10 | 228 | 183 |
| Generation 2.0 - Africa North | 204 | 277 | 481 | 0.03 | 64 | 91 |
| Generation 2.0 - India | 50 | 68 | 118 | 0.01 | 13 | 10 |
| Generation 2.0 - All Other | 10105 | 11171 | 21276 | 1.35 | 2604 | 2825 |
| Generation 2.5 - Mother Migrant - Nordic | 17403 | 18269 | 35672 | 2.27 | 4961 | 5135 |
| Generation 2.5 - Mother Migrant - Poland | 897 | 1102 | 1999 | 0.13 | 204 | 281 |
| Generation 2.5 - Mother Migrant - Turkey | 27 | 28 | 55 | < 0.01 | < 10 | < 10 |
| Generation 2.5 - Mother Migrant - Europe South | 464 | 520 | 984 | 0.06 | 136 | 128 |
| Generation 2.5 - Mother Migrant - Africa North | 30 | 39 | 69 | < 0.01 | < 10 | < 10 |
| Generation 2.5 - Mother Migrant - India | 63 | 62 | 125 | 0.01 | 11 | 18 |
| Generation 2.5 - Mother Migrant - All Other | 7376 | 7341 | 14717 | 0.94 | 1954 | 1868 |
| Generation 2.5 - Father Migrant - Nordic | 12635 | 13962 | 26597 | 1.69 | 3514 | 4092 |
| Generation 2.5 - Father Migrant - Poland | 377 | 401 | 778 | 0.05 | 97 | 107 |
| Generation 2.5 - Father Migrant - Turkey | 292 | 377 | 669 | 0.04 | 94 | 100 |
| Generation 2.5 - Father Migrant - Europe South | 2054 | 2306 | 4360 | 0.28 | 592 | 609 |
| Generation 2.5 - Father Migrant - Africa North | 541 | 681 | 1222 | 0.08 | 152 | 206 |
| Generation 2.5 - Father Migrant - India | 143 | 137 | 280 | 0.02 | 35 | 47 |
| Generation 2.5 - Father Migrant - All Other | 11750 | 12538 | 24288 | 1.54 | 3150 | 3371 |

**S-8A Table:** Results of multivariate cox proportional hazards models for transitions to third birth for females, differentiated by country-of-origin background.

| **Parameter** | **Hazard Ratio** | **CI 95lower** | **CI 95upper** |
| --- | --- | --- | --- |
| Generation 1.0 - Nordic (Ref: Native Swedish) | 1.13 | 1.08 | 1.19 |
| Generation 1.0 - Poland | 0.68 | 0.62 | 0.74 |
| Generation 1.0 - Turkey | 1.22 | 1.16 | 1.29 |
| Generation 1.0 - Europe South | 1.00 | 0.87 | 1.14 |
| Generation 1.0 - Africa North | 1.97 | 1.87 | 2.08 |
| Generation 1.0 - India | 0.75 | 0.64 | 0.88 |
| Generation 1.0 - All Other | 1.40 | 1.38 | 1.42 |
| Generation 1.5 - Nordic | 1.03 | 0.98 | 1.08 |
| Generation 1.5 - Poland | 0.93 | 0.84 | 1.03 |
| Generation 1.5 - Turkey | 1.64 | 1.54 | 1.75 |
| Generation 1.5 - Europe South | 1.00 | 0.83 | 1.21 |
| Generation 1.5 - Africa North | 1.57 | 1.26 | 1.95 |
| Generation 1.5 - India | 0.90 | 0.81 | 0.99 |
| Generation 1.5 - All Other | 1.18 | 1.16 | 1.21 |
| Generation 2.0 - Nordic | 1.02 | 0.99 | 1.05 |
| Generation 2.0 - Poland | 0.77 | 0.65 | 0.91 |
| Generation 2.0 - Turkey | 1.52 | 1.42 | 1.61 |
| Generation 2.0 - Europe South | 0.88 | 0.76 | 1.02 |
| Generation 2.0 - Africa North | 1.51 | 1.23 | 1.85 |
| Generation 2.0 - India | 0.83 | 0.45 | 1.55 |
| Generation 2.0 - All Other | 1.01 | 0.97 | 1.05 |
| Generation 2.5 - Mother Migrant - Nordic | 1.04 | 1.01 | 1.06 |
| Generation 2.5 - Mother Migrant - Poland | 1.07 | 0.95 | 1.20 |
| Generation 2.5 - Mother Migrant - Turkey | 1.21 | 0.61 | 2.42 |
| Generation 2.5 - Mother Migrant - Europe South | 0.99 | 0.83 | 1.18 |
| Generation 2.5 - Mother Migrant - Africa North | 0.85 | 0.40 | 1.78 |
| Generation 2.5 - Mother Migrant - India | 1.58 | 1.00 | 2.51 |
| Generation 2.5 - Mother Migrant - All Other | 1.08 | 1.03 | 1.13 |
| Generation 2.5 - Father Migrant - Nordic | 1.04 | 1.01 | 1.08 |
| Generation 2.5 - Father Migrant - Poland | 1.03 | 0.85 | 1.25 |
| Generation 2.5 - Father Migrant - Turkey | 0.98 | 0.81 | 1.19 |
| Generation 2.5 - Father Migrant - Europe South | 1.06 | 0.98 | 1.15 |
| Generation 2.5 - Father Migrant - Africa North | 1.25 | 1.09 | 1.43 |
| Generation 2.5 - Father Migrant - India | 1.48 | 1.11 | 1.97 |
| Generation 2.5 - Father Migrant - All Other | 1.08 | 1.05 | 1.12 |
| 1995-1998 (Ref: 1991-1994) | 0.73 | 0.68 | 0.77 |
| 1999-2002 | 0.84 | 0.79 | 0.89 |
| 2003-2006 | 0.99 | 0.94 | 1.06 |
| 2007-2010 | 1.05 | 0.99 | 1.12 |
| 2011-2014 | 1.01 | 0.95 | 1.07 |
| 2015-2017 | 0.99 | 0.93 | 1.05 |
| Age at Previous Birth | 0.88 | 0.87 | 0.88 |
| Education Secondary (Ref: Primary) | 0.88 | 0.86 | 0.89 |
| Education Tertiary | 1.20 | 1.18 | 1.22 |
| Education Missing | 1.34 | 1.29 | 1.40 |
| Unemployment Benefits: Yes (Ref: No) | 0.91 | 0.90 | 0.92 |
| Student Benefits: Yes (Ref: No) | 0.59 | 0.58 | 0.60 |
| In Employment: No (Ref: Yes) | 1.28 | 1.27 | 1.30 |

**S-8B Table:** Results of multivariate cox proportional hazards models for transitions to third birth for males, differentiated by country-of-origin background.

| **Parameter** | **Hazard Ratio** | **CI 95lower** | **CI 95upper** |
| --- | --- | --- | --- |
| Generation 1.0 - Nordic (Ref: Native Swedish) | 1.05 | 0.99 | 1.11 |
| Generation 1.0 - Poland | 0.78 | 0.70 | 0.86 |
| Generation 1.0 - Turkey | 1.59 | 1.52 | 1.66 |
| Generation 1.0 - Europe South | 1.02 | 0.93 | 1.13 |
| Generation 1.0 - Africa North | 3.07 | 2.92 | 3.23 |
| Generation 1.0 - India | 1.11 | 0.95 | 1.29 |
| Generation 1.0 - All Other | 1.91 | 1.88 | 1.93 |
| Generation 1.5 - Nordic | 1.07 | 1.02 | 1.12 |
| Generation 1.5 - Poland | 0.97 | 0.87 | 1.08 |
| Generation 1.5 - Turkey | 1.97 | 1.86 | 2.09 |
| Generation 1.5 - Europe South | 1.19 | 1.03 | 1.38 |
| Generation 1.5 - Africa North | 1.70 | 1.36 | 2.13 |
| Generation 1.5 - India | 1.00 | 0.85 | 1.17 |
| Generation 1.5 - All Other | 1.41 | 1.38 | 1.44 |
| Generation 2.0 - Nordic | 1.04 | 1.01 | 1.08 |
| Generation 2.0 - Poland | 0.96 | 0.80 | 1.14 |
| Generation 2.0 - Turkey | 1.63 | 1.51 | 1.75 |
| Generation 2.0 - Europe South | 1.01 | 0.89 | 1.15 |
| Generation 2.0 - Africa North | 1.72 | 1.34 | 2.19 |
| Generation 2.0 - India | 1.52 | 0.88 | 2.62 |
| Generation 2.0 - All Other | 1.04 | 1.00 | 1.08 |
| Generation 2.5 - Mother Migrant - Nordic | 1.07 | 1.04 | 1.10 |
| Generation 2.5 - Mother Migrant - Poland | 1.00 | 0.88 | 1.15 |
| Generation 2.5 - Mother Migrant - Turkey | 0.56 | 0.18 | 1.75 |
| Generation 2.5 - Mother Migrant - Europe South | 1.13 | 0.95 | 1.33 |
| Generation 2.5 - Mother Migrant - Africa North | 0.88 | 0.42 | 1.84 |
| Generation 2.5 - Mother Migrant - India | 0.79 | 0.44 | 1.42 |
| Generation 2.5 - Mother Migrant - All Other | 1.05 | 1.00 | 1.10 |
| Generation 2.5 - Father Migrant - Nordic | 1.02 | 0.98 | 1.05 |
| Generation 2.5 - Father Migrant - Poland | 1.05 | 0.86 | 1.29 |
| Generation 2.5 - Father Migrant - Turkey | 1.25 | 1.02 | 1.53 |
| Generation 2.5 - Father Migrant - Europe South | 1.14 | 1.05 | 1.23 |
| Generation 2.5 - Father Migrant - Africa North | 1.19 | 1.01 | 1.39 |
| Generation 2.5 - Father Migrant - India | 1.07 | 0.77 | 1.49 |
| Generation 2.5 - Father Migrant - All Other | 1.04 | 1.00 | 1.08 |
| 1995-1998 (Ref: 1991-1994) | 0.67 | 0.63 | 0.71 |
| 1999-2002 | 0.73 | 0.68 | 0.77 |
| 2003-2006 | 0.85 | 0.80 | 0.90 |
| 2007-2010 | 0.90 | 0.84 | 0.95 |
| 2011-2014 | 0.87 | 0.82 | 0.92 |
| 2015-2017 | 0.85 | 0.80 | 0.91 |
| Age at Previous Birth | 0.92 | 0.92 | 0.92 |
| Education Secondary (Ref: Primary) | 0.84 | 0.83 | 0.86 |
| Education Tertiary | 1.01 | 1.00 | 1.02 |
| Education Missing | 1.00 | 0.94 | 1.06 |
| Unemployment Benefits: Yes (Ref: No) | 1.07 | 1.05 | 1.08 |
| Student Benefits: Yes (Ref: No) | 0.99 | 0.96 | 1.01 |
| In Employment: No (Ref: Yes) | 1.18 | 1.16 | 1.19 |

**S-9 Table:** Overview of sociodemographic characteristics for the study population at time of third birth.

| **Characteristic** | **N** | **in %** |
| --- | --- | --- |
| **Sex** |  |  |
| Males | 216,402 | 49.19 |
| Females | 223,515 | 50.81 |
| **Population Subgroup** |  |  |
| Native Swedish | 306,749 | 69.73 |
| Generation 1.0 | 62,727 | 14.26 |
| Generation 1.5 | 24,547 | 5.58 |
| Generation 2.0 | 15,007 | 3.41 |
| Generation 2.5 - Mother Migrant | 14,721 | 3.35 |
| Generation 2.5 - Father Migrant | 16,166 | 3.67 |
| **Birth Cohorts** |  |  |
| 1940-1949 | 101 | 0.02 |
| 1950-1959 | 8,105 | 1.84 |
| 1960-1969 | 122,196 | 27.78 |
| 1970-1979 | 214,923 | 48.86 |
| 1980-1989 | 90,011 | 20.46 |
| 1990-1999 | 4,581 | 1.04 |
| Age in years: Median (IQR) | 34.26 | (30.89 - 37.49) |
| **Education** |  |  |
| Education Primary | 58,361 | 13.27 |
| Education Secondary | 206,183 | 46.87 |
| Education Tertiary | 171,648 | 39.02 |
| Education Missing | 3,725 | 0.85 |
| **Benefits** |  |  |
| Student No | 413,447 | 93.98 |
| Student Yes | 26,470 | 6.02 |
| Unemployed No | 389,580 | 88.56 |
| Unemployed Yes | 50,337 | 11.44 |
| **Employment** |  |  |
| Employed Yes | 363,480 | 82.62 |
| Employed No | 76,437 | 17.38 |

## Generation 1.0 – “Other” Category

**S-10A Table: Country of origin for migrants of generation 1.0 who were at risk of first birth – disaggregation of “other” category.**

| **Country of Origin** | **N Male** | **% Men** | **N Women** | **% Women** |
| --- | --- | --- | --- | --- |
| Bosnia Herzegovina | 11,371 | 0.03 | 9,017 | 3.07 |
| Yugoslavia | 21,499 | 5.63 | 16,480 | 5.60 |
| UK and Ireland | 17,161 | 4.50 | 6,545 | 2.23 |
| Germanic states | 17,705 | 4.64 | 16,623 | 5.65 |
| Netherlands | 4,521 | 1.18 | 3,005 | 1.02 |
| Latvia and Lithuania | 8,739 | 2.29 | 6,273 | 2.13 |
| Eastern Europe 1* | 6,974 | 1.83 | 13,619 | 4.63 |
| Bulgaria | 3,354 | 0.88 | 2,400 | 0.82 |
| Romania | 10,257 | 2.69 | 7,908 | 2.69 |
| Czech R and Slovakia | 2,432 | 0.64 | 2,486 | 0.85 |
| Hungary | 4,249 | 1.11 | 3,666 | 1.25 |
| France and Benelux | 8,531 | 2.24 | 5,220 | 1.78 |
| USA and Canada | 12,393 | 3.25 | 10,415 | 3.54 |
| C America and Caribbean | 4,567 | 1.20 | 3,810 | 1.30 |
| Chile | 4,009 | 1.05 | 2,715 | 0.92 |
| South America | 6,818 | 1.79 | 6,460 | 2.20 |
| Somalia and Djibouti | 13,883 | 3.64 | 11,326 | 3.85 |
| Eritrea | 14,700 | 3.85 | 8,439 | 2.87 |
| Ethiopia | 6,461 | 1.69 | 4,952 | 1.68 |
| Other Africa | 18,080 | 4.74 | 10,893 | 3.70 |
| Other Middle East | 7,294 | 1.91 | 3,581 | 1.22 |
| Lebanon | 7,961 | 2.09 | 4,858 | 1.65 |
| Syria | 40,579 | 10.64 | 16,392 | 5.57 |
| Iran | 21,478 | 5.63 | 16,721 | 5.69 |
| Iraq | 35,740 | 9.37 | 23,756 | 8.08 |
| China (excl. Taiwan and HK) | 13,465 | 3.53 | 14,786 | 5.03 |
| Other East Asia | 2,413 | 0.63 | 4,160 | 1.41 |
| Other South-East Asia / Pacific | 1,793 | 0.47 | 2,386 | 0.81 |
| Philippines | 1,088 | 0.29 | 6,148 | 2.09 |
| Vietnam | 3,551 | 0.93 | 4,215 | 1.43 |
| NZ and Australia | 4,896 | 1.28 | 2,371 | 0.81 |
| Thailand | 2,764 | 0.72 | 17,383 | 5.91 |
| Afghanistan | 12,623 | 3.31 | 5,022 | 1.71 |
| Pakistan and Bangladesh | 14,253 | 3.74 | 5,618 | 1.91 |
| Sri Lanka | 1,446 | 0.38 | 1,476 | 0.50 |
| North and South Korea | 698 | 0.18 | 1,289 | 0.44 |
| Eastern Europe 2** | 3,573 | 0.94 | 3,016 | 1.03 |
| Estonia | 2,765 | 0.72 | 2,889 | 0.98 |
| Brazil | 2,297 | 0.60 | 3,643 | 1.24 |
| Other | 3,159 | 0.83 | 2,078 | 0.71 |
| **Total** | **381,540** | **100.00** | **294,040** | **100.00** |

**S-10B Table: Country of origin for migrants of generation 1.0 who were at risk of second birth – disaggregation of “other” category.**

| **Country of Origin** | **N Male** | **% Men** | **N Women** | **% Women** |
| --- | --- | --- | --- | --- |
| Bosnia Herzegovina | 6,702 | 5.72 | 5,510 | 4.47 |
| Yugoslavia | 10,771 | 9.19 | 9,232 | 7.48 |
| UK and Ireland | 5,224 | 4.46 | 1,578 | 1.28 |
| Germanic states | 3,505 | 2.99 | 3,963 | 3.21 |
| Netherlands | 1,170 | 1.00 | 741 | 0.60 |
| Latvia and Lithuania | 1,242 | 1.06 | 2,016 | 1.63 |
| Eastern Europe 1* | 1,582 | 1.35 | 5,212 | 4.22 |
| Bulgaria | 633 | 0.54 | 856 | 0.69 |
| Romania | 2,220 | 1.90 | 2,762 | 2.24 |
| Czech R and Slovakia | 382 | 0.33 | 749 | 0.61 |
| Hungary | 712 | 0.61 | 1,020 | 0.83 |
| France and Benelux | 1,715 | 1.46 | 1,164 | 0.94 |
| USA and Canada | 2,821 | 2.41 | 2,365 | 1.92 |
| C America and Caribbean | 1,575 | 1.34 | 1,498 | 1.21 |
| Chile | 1,900 | 1.62 | 1,293 | 1.05 |
| South America | 2,339 | 2.00 | 2,659 | 2.16 |
| Somalia and Djibouti | 4,843 | 4.13 | 5,925 | 4.80 |
| Eritrea | 2,593 | 2.21 | 3,167 | 2.57 |
| Ethiopia | 2,505 | 2.14 | 2,476 | 2.01 |
| Other Africa | 5,899 | 5.04 | 4,796 | 3.89 |
| Other Middle East | 2,167 | 1.85 | 1,849 | 1.50 |
| Lebanon | 4,960 | 4.23 | 3,426 | 2.78 |
| Syria | 6,332 | 5.41 | 6,331 | 5.13 |
| Iran | 8,210 | 7.01 | 7,681 | 6.23 |
| Iraq | 18,823 | 16.07 | 16,244 | 13.17 |
| China (excl. Taiwan and HK) | 2,134 | 1.82 | 4,014 | 3.25 |
| Other East Asia | 344 | 0.29 | 1,198 | 0.97 |
| Other South-East Asia / Pacific | 540 | 0.46 | 992 | 0.80 |
| Philippines | 326 | 0.28 | 3,029 | 2.46 |
| Vietnam | 1,514 | 1.29 | 2,213 | 1.79 |
| NZ and Australia | 1,345 | 1.15 | 517 | 0.42 |
| Thailand | 505 | 0.43 | 5,613 | 4.55 |
| Afghanistan | 2,601 | 2.22 | 2,272 | 1.84 |
| Pakistan and Bangladesh | 3,479 | 2.97 | 3,330 | 2.70 |
| Sri Lanka | 645 | 0.55 | 814 | 0.66 |
| North and South Korea | 129 | 0.11 | 326 | 0.26 |
| Eastern Europe 2* | 940 | 0.80 | 1,283 | 1.04 |
| Estonia | 298 | 0.25 | 1,076 | 0.87 |
| Brazil | 517 | 0.44 | 1,351 | 1.10 |
| Other | 1,001 | 0.85 | 824 | 0.67 |
| **Total** | **117,143** | **100.00** | **123,365** | **100.00** |

**S-10C Table:** Country of origin for migrants of generation 1.0 who were at risk of third birth – disaggregation of “other” category.

| **Country of Origin** | **N Male** | **% Men** | **N Women** | **% Women** |
| --- | --- | --- | --- | --- |
| Bosnia Herzegovina | 4,748 | 6.52 | 3,744 | 5.18 |
| Yugoslavia | 7,534 | 10.35 | 6,288 | 8.71 |
| UK and Ireland | 3,120 | 4.29 | 861 | 1.19 |
| Germanic states | 1,930 | 2.65 | 2,228 | 3.08 |
| Netherlands | 690 | 0.95 | 414 | 0.57 |
| Latvia and Lithuania | 427 | 0.59 | 908 | 1.26 |
| Eastern Europe 1* | 689 | 0.95 | 2,326 | 3.22 |
| Bulgaria | 291 | 0.40 | 375 | 0.52 |
| Romania | 968 | 1.33 | 1,205 | 1.67 |
| Czech R and Slovakia | 186 | 0.26 | 394 | 0.55 |
| Hungary | 314 | 0.43 | 446 | 0.62 |
| France and Benelux | 967 | 1.33 | 624 | 0.86 |
| USA and Canada | 1,528 | 2.10 | 1,198 | 1.66 |
| C America and Caribbean | 838 | 1.15 | 787 | 1.09 |
| Chile | 1,180 | 1.62 | 767 | 1.06 |
| South America | 1,272 | 1.75 | 1,344 | 1.86 |
| Somalia and Djibouti | 3,616 | 4.97 | 4,366 | 6.04 |
| Eritrea | 1,430 | 1.96 | 1,570 | 2.17 |
| Ethiopia | 1,838 | 2.52 | 1,649 | 2.28 |
| Other Africa | 3,277 | 4.50 | 2,725 | 3.77 |
| Other Middle East | 1,376 | 1.89 | 1,245 | 1.72 |
| Lebanon | 3,957 | 5.44 | 2,732 | 3.78 |
| Syria | 3,437 | 4.72 | 3,619 | 5.01 |
| Iran | 4,695 | 6.45 | 3,971 | 5.50 |
| Iraq | 13,641 | 18.74 | 12,117 | 16.78 |
| China (excl. Taiwan and HK) | 884 | 1.21 | 1,741 | 2.41 |
| Other East Asia | 126 | 0.17 | 538 | 0.74 |
| Other South-East Asia / Pacific | 319 | 0.44 | 516 | 0.71 |
| Philippines | 175 | 0.24 | 1,400 | 1.94 |
| Vietnam | 1,065 | 1.46 | 1,422 | 1.97 |
| NZ and Australia | 788 | 1.08 | 262 | 0.36 |
| Thailand | 241 | 0.33 | 2,262 | 3.13 |
| Afghanistan | 1,511 | 2.08 | 1,392 | 1.93 |
| Pakistan and Bangladesh | 1,917 | 2.63 | 1,905 | 2.64 |
| Sri Lanka | 386 | 0.53 | 490 | 0.68 |
| North and South Korea | 41 | 0.06 | 133 | 0.18 |
| Eastern Europe 2** | 496 | 0.68 | 657 | 0.91 |
| Estonia | 128 | 0.18 | 604 | 0.84 |
| Brazil | 238 | 0.33 | 579 | 0.80 |
| Other | 540 | 0.74 | 424 | 0.59 |
| **Total** | **72,804** | **100.00** | **72,228** | **100.00** |

* Eastern Europe 1: Moldova, Russia, USSR, Ukraine, Belarus

** Eastern Europe 2: Armeina, Azerbeijan, Georgia, Kazakhstan, Kyrgyzstan, Tajikistan, Turkmenistan, Uzbekistan

## Disaggregation of “Nordic” Category into Finns, Danes, and Norwegians

**S-11a Table:** Disaggregation of the Generation 1.0 “Nordic” group into individuals from Finland, Denmark, and Norway.

| **Event** | **G1.0 “Nordic”** | **G1.0 Finland** | **G1.0 Denmark** | **G1.0 Norway** |
| --- | --- | --- | --- | --- |
| 1st birth: Females (0,2] | 1.08 (1.05-1.11) | 0.74 (0.70-0.78) | 1.73 (1.65-1.82) | 1.05 (0.99-1.12) |
| 1st birth: Females (2,5] | 1.17 (1.14-1.21) | 0.85 (0.80-0.89) | 1.81 (1.70-1.91) | 1.32 (1.25-1.39) |
| 1st birth: Females (2,Inf] | 0.86 (0.83-0.88) | 0.77 (0.75-0.80) | 1.07 (0.99-1.17) | 1.03 (0.97-1.09) |
| 2nd birth: Females | 0.93 (0.90-0.95) | 0.91 (0.88-0.94) | 0.91 (0.86-0.95) | 0.99 (0.95-1.04) |
| 3rd birth: Females | 1.13 (1.08-1.19) | 1.18 (1.10-1.27) | 1.02 (0.92-1.14) | 1.14 (1.04-1.24) |
| 1st birth: Males (0,2] | 1.63 (1.57-1.68) | 1.08 (1.00-1.16) | 2.12 (2.03-2.23) | 1.52 (1.42-1.61) |
| 1st birth: Males (2,5] | 1.40 (1.35-1.45) | 1.05 (0.98-1.13) | 1.78 (1.68-1.88) | 1.34 (1.27-1.43) |
| 1st birth: Males (2,Inf] | 0.84 (0.82-0.87) | 0.64 (0.61-0.67) | 1.18 (1.11-1.26) | 1.09 (1.03-1.15) |
| 2nd birth: Males | 0.95 (0.93-0.98) | 0.90 (0.86-0.95) | 1.02 (0.98-1.07) | 0.93 (0.89-0.98) |
| 3rd birth: Males | 1.05 (0.99-1.11) | 1.12 (1.01-1.24) | 0.95 (0.85-1.05) | 1.08 (0.98-1.18) |

**S-11b Table:** Disaggregation of the Generation 1.5 “Nordic” group into individuals from Finland, Denmark, and Norway.

| **Event** | **G1.5 “Nordic”** | **G1.5 Finland** | **G1.5 Denmark** | **G1.5 Norway** |
| --- | --- | --- | --- | --- |
| 1st birth: Females | 0.97 (0.95-0.99) | 0.93 (0.90-0.96) | 1.02 (0.96-1.08) | 1.06 (1.00-1.13) |
| 2nd birth: Females | 0.84 (0.82-0.87) | 0.82 (0.79-0.85) | 0.89 (0.83-0.95) | 0.87 (0.81-0.94) |
| 3rd birth: Females | 1.03 (0.98-1.08) | 0.99 (0.93-1.05) | 1.09 (0.96-1.23) | 1.13 (0.99-1.28) |
| 1st birth: Males | 0.91 (0.89-0.93) | 0.85 (0.83-0.88) | 1.12 (1.06-1.18) | 1.01 (0.95-1.08) |
| 2nd birth: Males | 0.89 (0.87-0.91) | 0.86 (0.83-0.89) | 1.00 (0.94-1.06) | 0.91 (0.84-0.98) |
| 3rd birth: Males | 1.07 (1.02-1.12) | 1.06 (1.00-1.12) | 1.05 (0.93-1.18) | 1.19 (1.04-1.37) |

**S-11c Table:** Disaggregation of the Generation 2.0 “Nordic” group into the individuals from Finland, Denmark, and Norway.

| **Event** | **G2.0 “Nordic”** | **G2.0 Finland** | **G2.0 Denmark** | **G2.0 Norway** |
| --- | --- | --- | --- | --- |
| 1st birth: Females | 1.04 (1.02-1.05) | 1.04 (1.02-1.06) | 1.03 (0.96-1.10) | 1.06 (0.97-1.16) |
| 2nd birth: Females | 0.91 (0.89-0.92) | 0.91 (0.89-0.92) | 0.92 (0.85-1.00) | 0.92 (0.82-1.02) |
| 3rd birth: Females | 1.02 (0.99-1.05) | 1.02 (0.98-1.05) | 0.96 (0.83-1.11) | 1.12 (0.91-1.38) |
| 1st birth: Males | 0.97 (0.95-0.98) | 0.96 (0.94-0.97) | 1.09 (1.02-1.16) | 1.11 (1.02-1.21) |
| 2nd birth: Males | 0.90 (0.88-0.92) | 0.89 (0.88-0.91) | 0.97 (0.90-1.04) | 1.06 (0.96-1.17) |
| 3rd birth: Males | 1.04 (1.01-1.08) | 1.04 (1.00-1.08) | 1.02 (0.89-1.17) | 1.14 (0.96-1.37) |

**S-11d Table:** Disaggregation of the "Generation 2.5 Mother Migrant “Nordic” group into the individuals from Finland, Denmark, and Norway.

| **Event** | **G2.5 “Nordic”** | **G2.5 Finland** | **G2.5 Denmark** | **G2.5 Norway** |
| --- | --- | --- | --- | --- |
| 1st birth: Females | 0.96 (0.95-0.97) | 0.94 (0.93-0.96) | 1.01 (0.97-1.05) | 1.01 (0.98-1.04) |
| 2nd birth: Females | 0.96 (0.94-0.97) | 0.96 (0.94-0.98) | 0.93 (0.89-0.97) | 0.95 (0.91-0.98) |
| 3rd birth: Females | 1.04 (1.01-1.06) | 1.04 (1.01-1.07) | 1.00 (0.92-1.08) | 1.03 (0.96-1.11) |
| 1st birth: Males | 0.93 (0.92-0.95) | 0.91 (0.90-0.92) | 1.03 (0.99-1.06) | 0.97 (0.95-1.00) |
| 2nd birth: Males | 0.94 (0.92-0.95) | 0.93 (0.92-0.95) | 0.96 (0.92-1.01) | 0.94 (0.91-0.98) |
| 3rd birth: Males | 1.07 (1.04-1.10) | 1.09 (1.05-1.12) | 1.02 (0.94-1.10) | 1.05 (0.98-1.12) |

**S-11e Table:** Disaggregation of the Generation 2.5 Father Migrant “Nordic” group into the individuals from Finland, Denmark, and Norway.

| **Event** | **G2.5 “Nordic”** | **G2.5 Finland** | **G2.5 Denmark** | **G2.5 Norway** |
| --- | --- | --- | --- | --- |
| 1st birth: Females | 1.04 (1.03-1.05) | 1.05 (1.03-1.07) | 1.02 (0.99-1.05) | 1.02 (0.99-1.06) |
| 2nd birth: Females | 0.92 (0.91-0.94) | 0.93 (0.91-0.95) | 0.92 (0.89-0.96) | 0.94 (0.90-0.98) |
| 3rd birth: Females | 1.04 (1.01-1.08) | 1.03 (0.99-1.07) | 1.04 (0.97-1.11) | 1.09 (1.01-1.18) |
| 1st birth: Males | 1.01 (0.99-1.02) | 1.00 (0.99-1.02) | 1.03 (1.00-1.06) | 0.98 (0.95-1.02) |
| 2nd birth: Males | 0.93 (0.91-0.94) | 0.92 (0.90-0.95) | 0.91 (0.88-0.95) | 0.93 (0.89-0.97) |
| 3rd birth: Males | 1.02 (0.98-1.05) | 0.99 (0.95-1.04) | 1.05 (0.98-1.12) | 1.04 (0.96-1.13) |
